# ArxA From *Azoarcus* sp. CIB, an Anaerobic Arsenite Oxidase From an Obligate Heterotrophic and Mesophilic Bacterium

**DOI:** 10.3389/fmicb.2019.01699

**Published:** 2019-07-30

**Authors:** Gonzalo Durante-Rodríguez, Helga Fernández-Llamosas, Elena Alonso-Fernandes, María Nieves Fernández-Muñiz, Riansares Muñoz-Olivas, Eduardo Díaz, Manuel Carmona

**Affiliations:** ^1^Biotecnología Microbiana y de Plantas, Centro de Investigaciones Biológicas-CSIC, Madrid, Spain; ^2^Departamento de Química Analítica, Facultad de Químicas, Universidad Complutense Madrid, Madrid, Spain

**Keywords:** arsenic, arsenite, *Azoarcus*, *arx* cluster, anaerobiosis

## Abstract

Arsenic is a toxic element widely distributed in nature, but numerous bacteria are able to resist its toxicity mainly through the *ars* genes encoding an arsenate reductase and an arsenite efflux pump. Some “arsenotrophic” bacteria are also able to use arsenite as energy supplier during autotrophic growth by coupling anaerobic arsenite oxidation *via* the *arx* gene products to nitrate respiration or photosynthesis. Here, we have demonstrated that *Azoarcus* sp. CIB, a facultative anaerobic β-proteobacterium, is able to resist arsenic oxyanions both under aerobic and anaerobic conditions. Genome mining, gene expression, and mutagenesis studies revealed the presence of a genomic island that harbors the *ars* and *arx* clusters involved in arsenic resistance in strain CIB. Orthologous *ars* clusters are widely distributed in the genomes of sequenced *Azoarcus* strains. Interestingly, genetic and metabolic approaches showed that the *arx* cluster of the CIB strain encodes an anaerobic arsenite oxidase also involved in the use of arsenite as energy source. Hence, *Azoarcus* sp. CIB represents the prototype of an obligate heterotrophic bacterium able to use arsenite as an extra-energy source for anaerobic cell growth. The arsenic island of strain CIB supports the notion that metabolic and energetic skills can be gained by genetic mobile elements.

## Introduction

Arsenic (As) is an element that is widely distributed in nature, either present naturally as an element of the soil composition or due to its release from anthropogenic sources. Arsenic is a toxic element for living organisms and is also a human carcinogen (Mead, 2005), ranking in first position in the Priority List of Hazardous Substances by the US Environmental Protection Agency. The arsenic toxicology depends on its oxidation state and its chemical forms. Thus, the oxidized arsenate [As(V)] is the prevalent form of arsenic in aerobic environments and is less toxic than the reduced arsenite [As(III)], which is most common form under anoxic conditions (Stolz and Oremland, 1999; Rosen, 2002). Resistance to arsenic is widely spread among bacteria, and different resistance strategies, e.g., As uptake selectivity, As(III) oxidation, As(V) reduction, efflux of all As compounds, As methylation and volatilization, have been reported (Zhu et al., 2014). The molecular basis of some of the mechanisms of arsenic resistance has been well-studied (Mukhopadhyay et al., 2002; Rosen, 2002). The most common and widely distributed As resistance mechanism, i.e., arsenate reduction coupled to arsenite extrusion, is encoded by the *ars* genes present in many bacteria and archaea, where they show remarkable diversity in their sequence and genomic organization (Mukhopadhyay and Rosen, 2002), and they can be plasmid borne or chromosomally encoded (Oden et al., 1994; Cai et al., 1998).

It has been already described that arsenite is much more toxic than arsenate and, therefore, bacteria are usually able to resist 100-fold higher concentrations of arsenate than arsenite (Williams and Silver, 1984). To increase the resistance to arsenite, some bacteria have, in addition to the *ars* genes that encode an arsenite efflux pump, a gene cluster that encodes an arsenite oxidase able to transform arsenite to the less toxic arsenate (Stolz et al., 2010). Two classes of bacterial arsenite oxidases have been described until now: (1) the aerobic AioAB that transfers electrons arising from the oxidation of As(III) toward a periplasmic soluble electron carrier ultimately reducing O_2_ (Anderson et al., 1992), and (2) ArxAB that catalyzes an anaerobic oxidation of As(III) (Zargar et al., 2012). Aerobic arsenite oxidation was first described in 1918 (Green, 1918), but their biological relevance was not understood until 1949 when 15 heterotrophic bacteria with the ability to oxidize arsenite to arsenate were isolated (Turner, 1949). Since then, a good number of chemolithotrophic and autotrophic bacteria have also been isolated (Oremland and Stolz, 2005; Yamamura and Amachi, 2014). Whereas the heterotrophic arsenite oxidation is generally assumed to be a detoxification process in which the microorganisms do not obtain energy from the oxidation of As(III) (Ehrlich, 2002; Silver and Phung, 2005), autotrophic strains are able to derive reducing power and energy from the oxidation of arsenite for carbon dioxide (CO_2_) fixation (Silver and Phung, 2005).

The anaerobic oxidation of arsenite is the more recent mechanism of arsenotrophy described in chemotrophic and autotrophic bacteria (Oremland et al., 2017). The anaerobic arsenite oxidation has been linked to the *arx* genes present in the extremophiles *Alkalilimnicola ehrlichii* MLHE-1 (Zargar et al., 2010, 2012) and *Ectothiorhodospira* sp. BSL9 (Hernández-Maldonado et al., 2017). *A. ehrlichii* MLHE-1 and *Ectothiorhodospira* sp. BSL9 are able to couple the arsenite oxidation to anaerobic nitrate respiration or to an anaerobic photosynthesis with CO_2_ fixation, respectively (Hernández-Maldonado et al., 2017; Oremland et al., 2017). The analysis of the *A. ehrilichii* MLHE-1 genome found two genes, *arxA* and *arxB*, that coded for the putative ArxA and ArxB subunits of a molybdopterin-oxidoreductase belonging to the dimethyl sulfoxide (DMSO) reductase family with arsenite oxidase activity (Zargar et al., 2012). Interestingly, ArxAB showed higher amino acid sequence identity with the respiratory arsenate reductase ArrAB than with the aerobic arsenite oxidase AioAB (Zargar et al., 2012; Van Lis et al., 2013).The *arx* cluster of *A. ehrilichii* MLHE-1 includes the *arxXRS* genes, which encode regulatory proteins, and the *arxB2ABCD* genes, which encode the structural components of the putative arsenite oxidase complex: ArxB2, a [4Fe-4S] containing protein; ArxA, the large subunit of the arsenite oxidase; ArxB, a [4Fe-4S] containing reductase; ArxC, a membrane protein able to oxidize quinol groups; and ArxD, one TorD-like chaperone protein (Zargar et al., 2012). The *arx* gene cluster is highly conserved in all extremophiles isolated from As-rich environments or other extreme environments, such *Thiocapsa* spp., *Thioalkalivibrio* spp. or *Halomonas* spp., and also in the metagenomic samples analyzed (Andres and Bertin, 2016; Oremland et al., 2017). However, the *arx* genes from bacteria other than the extremophiles cited above have not been studied so far, and the extra-energy derived from arsenite oxidation has been only reported in the autotrophic metabolism but not in heterotrophic anaerobes able to oxidize arsenite to arsenate (Hernández-Maldonado et al., 2017).

*Azoarcus* sp. CIB is a facultative anaerobe and obligate heterotrophic β-proteobacterium, able to degrade a high number of organic compounds, including aromatics under aerobic and/or anaerobic (denitrifying) conditions (López-Barragán et al., 2004; Carmona et al., 2009; Juárez et al., 2013; Blázquez et al., 2018). In addition, the CIB strain can associate with plants living as an endophyte in the root of rice (Fernández-Llamosas et al., 2014). Genome mining in *Azoarcus* sp. CIB evidenced the presence of a high number of gene clusters encoding potential resistance to heavy metals (Supplementary Table S1; Martín-Moldes et al., 2015). It has been already demonstrated that *Azoarcus* sp. CIB is resistant to moderate concentrations of selenite (Fernández-Llamosas et al., 2016) and our results also showed resistance to tellurite, zinc, cadmium, and nickel (data not shown). Thus, the strain CIB is an environmentally relevant and a promising microorganism to treat samples contaminated with aromatic compounds and metals due to the high number of putative solvent degradation and heavy metals gene clusters that has been identified in their genome. It has been already demonstrated that *Azoarcus* sp. CIB is resistant to moderate concentrations of selenite (Fernández-Llamosas et al., 2016), and our results also showed resistance to tellurite, zinc, cadmium, and nickel (data not shown). Since arsenic is one of the most important environmental toxic compounds and there is scared knowledge about the resistance capacity of bacteria of the genus *Azoarcus* towards this element, we analyzed here the ability of the strain CIB to resist arsenite and arsenate. *Azoarcus* sp. CIB carries a genomic island likely acquired through horizontal gene transfer that harbors the *ars* and the *arx* clusters involved in arsenic resistance. Moreover, we provide experimental evidence that the *arx* genes allow *Azoarcus* sp. CIB to oxidize arsenite under anaerobic conditions, hence constituting the first obligate heterotrophic non-extremophile bacterium described so far able to utilize the electrons from arsenite oxidation as an extra-energy for cell growth.

## Materials and Methods

### Bacterial Strains, Plasmids, and Growth Conditions

Bacterial strains and plasmids used in this work are detailed in Table 1. *Azoarcus* sp. strain CIB was deposited in the Spanish Type Culture Collection (CECT #5669). *Azoarcus* strains were grown on MC medium (MA basal medium plus trace elements and vitamins). MA basal medium is composed of the following, per liter of distillated water: 0.33 g of KH_2_PO_4_, 1.2 g of Na_2_HPO_4_, 0.11 g of NH_4_Cl, 0.1 g MgSO_4_ × 7H_2_O, 0.04 g of CaCl_2_ (pH 7.5) supplemented with trace elements [stock solution 100×; 1.5 g of nitrilotriacetic acid, 3 g of MgSO_4_ × 7H_2_O, 0.5 g of MnSO_4_ × 2H_2_O, 1 g of NaCl, 0.1 g of FeSO_4_ × 7H_2_O, 0.18 g of CoSO_4_ × 7H_2_O, 0.1 g of CaCl_2_ × 2H_2_O, 0.18 g of ZnSO_4_ × 7H_2_O, 0.01 g of CuSO_4_ × 5H_2_O, 0.02 g of KAl(SO_4_)_2_ × 12H_2_O, 0.01 g of H_3_BO_3_, 0.01 g of Na_2_MoO × 2H_2_O, 0.025 g of NiCl_2_ × 6H_2_O, and 0.3 mg of Na_2_ScO_3_ × 5H_2_O (pH 6.5) per liter of deionized water], vitamin solution (stock 1,000×; 20 mg of biotin, 20 mg of folic acid, 10 mg of pyridoxine-HCl, 50 mg of thiamine-HCl × 2H_2_O, 50 mg of riboflavin, 50 mg of nicotinic acid, 50 mg of calcium *D*-pantothenic acid, 50 mg of vitamin B12, and 50 mg of *p*-aminobenzoic acid per liter of distilled water) (López-Barragán et al., 2004). For anaerobic growth, 15 ml of MC medium was flushed with N_2_, and the bottles were sealed with rubber stoppers and aluminum crimp seals before being autoclaved, and 10 mM potassium nitrate was added as electron acceptor (López-Barragán et al., 2004). As carbon source, 0.2% (w/v) pyruvate was added. For anaerobic growth conditions, the carbon source and the bacterial inoculum were injected through the stopper with a syringe. All the cultures were incubated at 30°C. *E. coli* strains were grown in lysogeny broth (LB) medium (Miller, 1972) at 37°C. When required, kanamycin was added at 50 μg ml^−1^. Growth was monitored by measuring the absorbance at 600 nm (*A*_600_).

**Table 1:** Bacterial strains and plasmids used in this study.

Strain or plasmid	Relevant genotype and characteristic(s)	Reference or source
*E. coli* strains		
DH5α	*endA1 hsdR17 supE44 thi-1 recA1 gyrA*(Nal^r^) *relA1* Δ(*argF-lac*) *U169 depR* Φ*80dlacd(lacZ) M15*	Sambrook and Rusell, 2001
S17-1λ*pir*	Tp^r^ Sm^r^ *recA thi hsdRM^+^* RP4::2-Tc::Mu::Km λ*pir* phage lysogen	De Lorenzo and Timmis, 1994
*Azoarcus* strains		
CIB	Wild type strain	López-Barragán et al., 2004
CIBd*arsC*	*Azoarcus* sp. CIB with a disruption in the *arsC* gene	This work
CIBd*arsC2*	*Azoarcus* sp. CIB with a disruption in the *arsC2* gene	This work
CIBd*arsB*	*Azoarcus* sp. CIB with a disruption in the *arsB* gene	This work
CIBd*arxA*	*Azoarcus* sp. CIB with a disruption in the *arxA* gene	This work
Plasmids		
pK18*mob*	Km^r^, *ori*ColE1 Mob^+^ *lacZα*, used for directed insertional disruption	Schäfer et al., 1994
pK18*mobarsC*	Km^r^, 468 bp *arsC* internal fragment cloned in double-digested HindIII/BamHI pK18*mob*	This work
pK18*mobarsC2*	Km^r^, 551 bp *arsC2* internal fragment cloned in double-digested HindIII/BamHI pK18*mob*	This work
pK18*mobarsB*	Km^r^, 557 bp *arsB* internal fragment cloned in double-digested HindIII/BamHI pK18*mob*	This work
pK18*mobarxA*	Km^r^, 493 bp *arxA* internal fragment cloned in double-digested HindIII/BamHI pK18*mob*	This work

### Molecular Biology Techniques

Standard molecular biology techniques were performed as previously described (Sambrook and Rusell, 2001). DNA fragments were purified with Gene-Turbo (BIO101 Systems). Plasmids and PCR products were purified with a High Pure Plasmid and PCR Product Purifications kits (Roche), respectively. Oligonucleotides were supplied by Sigma Co, and they are detailed in Supplementary Table S2. All cloned inserts and DNA fragments were confirmed by DNA sequencing with fluorescently labeled dideoxynucleotide terminators (Sanger et al., 1977) and AmpliTaq FS DNA polymerase (Applied Biosystems) in an ABI Prism 377 automated DNA sequencer (Applied Biosystems). Transformations of *E. coli* were carried out by using the RbCl method or by electroporation (Gene Pulser, Bio-Rad) (Sambrook and Rusell, 2001).

### Construction of *Azoarcus* sp. CIBd*arsC*, *Azoarcus* sp. CIBd*arsC2*, *Azoarcus* sp. d*arsB*, and *Azoarcus* sp. CIBd*arxA* Mutant Strains

For insertional disruption of *arsC*, *arsC2*, *arsB*, and *arxA* genes in the genome of *Azoarcus* sp. CIB, we used single homologous recombination with PCR-amplified DNA fragments obtained with the primer pairs 5′arsC/3′arsC, 5′arsC2/3′arsC2, 5′arsB/3′arsB, and 5′arxA/3′arxA, respectively (Supplementary Table S2). The obtained fragments were double-digested with the appropriate restriction enzymes, generating the pK18mobarsC, pK18mobarsC2, pK18mobarsB, and pK18mobarxA recombinant plasmids (Supplementary Table S2). These plasmids were transferred from *E. coli* S17-1λ*pir* (donor strain) to *Azoarcus* sp. CIB (recipient strain) by biparental filter mating (De Lorenzo and Timmis, 1994), and exconjugants strains *Azoarcus* sp. CIBd*arsC*, *Azoarcus* sp. CIBd*arsC2, Azoarcus* sp. CIBd*arsB*, and *Azoarcus* sp. CIBd*arxA* was isolated aerobically on kanamycin-containing MC agar plates harboring 10 mM glutarate as the sole carbon source for counter selection of donor cells. The gene disruption through single homologous technique promote polar effects has been already stated (López-Barragán et al., 2004). For that reason we did not complement the mutant strains, instead the mutants were analyzed by PCR with the appropriate oligonucleotides (Supplementary Table S2) to confirm the disruption of the target genes (Supplementary Figure S15).

### RNA Extraction and Quantitative Reverse Transcription-PCR (qRT-PCR) Experiments

RNA was purified from bacterial cells grown up to the middle of the exponential phase in the conditions described in each experiment were resuspended in a solution containing TE buffer (Tris-HCl 10 mM pH 8.0, EDTA 1 mM) and lysozyme 50 mg/ml (Sigma). Total RNA was obtained using High Pure RNA Isolation Kit (Roche). The DNA was removed with DNAse and Removal Treatment Kit (Ambion). The concentration and purity of RNA was spectrophotometically determined at A_260_ and calculating the A_260_/A_280_ ratio, respectively. The cDNA was obtained by using the Transcriptor First Strand cDNA Synthesis kit (Roche). Each RT reaction (20 μl) contained 1 μg RNA, 10 U reverse transcriptase, RNAse inhibitor 20 U, dNTPS 1 mM and 60 μM random hexamer primers. The standard procedure of cDNA production includes a 10 min incubation at 25°C followed by 1 cycle of 30 min at 55°C and another incubation of 5 min at 85°C using the Mastercycler Gradient equipment (Eppendorf). Then, 1 μl of the obtained cDNA was used as template for the PCR. The cDNA was PCR-amplified using the required oligonucleotides at final concentration of 0.5 μM and 1 U of DNA polymerase I (Biotools) in a final volume of 50 μl. qRT-PCR was performed in a LightCycle®480 II Real-Time PCR Instrument (Roche). The volume of reaction was 20 μl and contained 1 μl cDNA, 0.25 μM oligonucleotides, and 10 μl SYBR Green Master Mix (Roche). The *dnaE* gene was used as housekeeping control as reported before (Valderrama et al., 2012). The results are shown as relative quantification using the ΔΔCt method (Livak and Schmittgen, 2001).

### Determination of Total Arsenic in Bacteria by ICP-MS

Bacterial extracts were digested by acid digestion in a 1,000 W microwave oven (MSP microwave oven, CEM, Matthews, NC, USA) with 750 μl of HNO_3_ and 250 μl of 30% (v/v) H_2_O_2_. (Scharlau, SPAIN). The resulting solutions were diluted by adding 5 ml of deionized water. Arsenic concentration was determined in an Agilent 7700 ICP-MS (Agilent Technologies, Santa Clara, CA, USA), equipped with a Conikal nebulizer, Fassel torch and double pass Scott-type spray chamber cooled by a Peltier system. The equipment measuring conditions are summarized in Supplementary Table S3.

### Determination of Arsenic Species in Bacterial Extracts by HPLC-ICP-MS

Inorganic arsenic species were extracted with 1:1 methanol:water using an ultrasonic homogenizer SONOPULS HD 2200 (30%, 60 s). Extracts were centrifuged (7,500*g*, 15 min) and supernatant filtered through a 0.22-μm nylon syringe before injection into the HPLC-ICP/MS for arsenic speciation. Diluted samples were injected onto an anion-exchange column PRP-X100 (250 × 4.1 mm, particle size 10 μm; Hamilton, Reno, NY). Arsenic species were eluted in 10 mM ${HPO}_{4}^{2-}/H_{2}{PO}_{4}^{1-}$, 2% (v/v) MeOH mobile phase, at 1.5 ml min^−1^ flow rate. Identification of As species was carried out by matching retention times and by spiking both species to a sample. As(III) and As(V) was quantified on an As(III) and As(V) calibration curve. Extraction recoveries obtained were in the range 85–100%. The experimental conditions are listed in Supplementary Table S3.

### Arsenate Reduction Experiments

The production of arsenite by resting cells of *Azoarcus* sp. CIB in the presence of 5 mM arsenate was checked by a colorimetric assay according to a previously described protocol (Watanabe et al., 2017). Briefly, 1 ml of CIB cultured cells aerobically grown for 72 h in MC medium supplemented with pyruvate 0.2% were pelleted and resuspended in fresh MC medium amended with pyruvate 0.02% and 5 mM arsenate and incubated at 30°C for 3 h. As negative control, 1 ml of culture cells were collected and boiled at 95°C for 5 min. The boiled cells were processed in the same resting conditions than the live culture. After the incubation, the culture was mixed with 40 μl of 10 mM DEDTC (diethyldithiocarbamate) prepared in 50% ethanol and 20 μl of 2.5 M nitric acid solution. After 10 min of incubation at room temperature, 20 μl of 2 mM Cu(NO_3_)_2_ was added to the mix and immediately filtered through a MCE filter with a 0.22 μm pore size. The colored complex As(III)-DEDTC was visualized in the filter.

### Bioluminescence-ATP Concentration Assay

The ATP concentration was measured using the commercial “ATP Biomass Kit HS” provided by BioThema AB (Handen, Sweden). Fifty microliter of each CIB cell culture were mixed with 50 μl of Extractant B/S solution to release the ATP from the cells. Then, the ATP was mixed with 400 μl of ATP-Reagent HS and the light emission from the firefly luciferase reaction was measured at 25°C in a Luminometer TD-20/20 (Turner Design). The light intensity was corrected by the cell growth in each case. The luciferase reaction follows first-order kinetics, and the ATP-Reagent HS in the kit produces light whose intensity was proportional to the ATP concentration (Lundin, 2000).

### Sequence Data Analyses

Nucleotide sequence analyses were done at the National Center for Biotechnology Information (NCBI) server. Pairwise and multiple protein sequence alignments were made with the ClustalW program (Thompson et al., 1994) at the EMBL-EBI server. Phylogenetic analysis of different proteins was carried out according to the Kimura two-parameter method (Kimura, 1980), and a tree was reconstructed using the neighbor-joining method (Saitou and Nei, 1987) of the PHYLIP program (Felsenstein, 1993).

## Results and Discussion

### The Arsenic Resistance of *Azoarcus* sp. CIB

**Figure 1:**
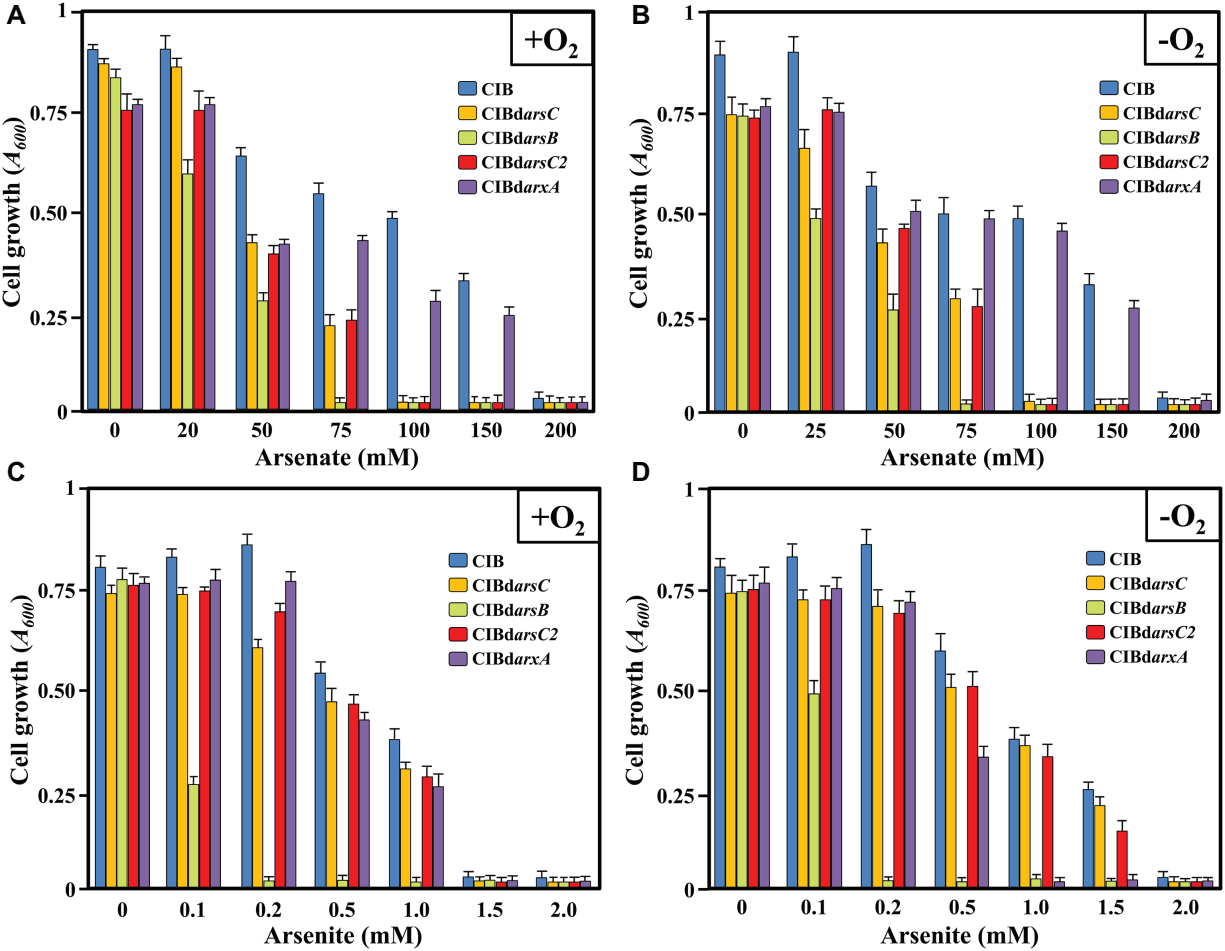
Cell growth of *Azoarcus* sp. CIB (CIB), *Azoarcus* sp. CIBd*arsC* (CIBd*arsC*), *Azoarcus* sp. CIBd*arsB* (CIBd*arsB*), *Azoarcus* sp. CIBd*arsC2* (CIBd*arsC2*), and *Azoarcus* CIBd*arxA* (CIBd*arxA*) expressed as absorbance at 600 nm (*A*_600_) at 72 h of incubation (end of exponential phase) in aerobic **(A,C)** or anaerobic **(B,D)** conditions, in the presence of increasing concentrations of arsenate **(A,B)** or arsenite **(C,D)**. Error bars indicate the standard deviations of three independent experiments. The inset shows the color code of the *Azoarcus* sp. CIB strains analyzed.

To investigate the ability of *Azoarcus* sp. CIB to tolerate arsenic in two of their ionic forms, cells were grown, either under aerobic or anaerobic (using 10 mM of NaNO_3_ as final electron acceptor) conditions, in minimal medium supplemented with 0.2% pyruvate (18 mM) as sole carbon source and increasing concentrations of [As(V)] or [As(III)]. Whereas the strain CIB was able to grow in the presence of arsenate concentrations as high as 150 mM, it did not grow at arsenite concentrations higher than 1 mM; similar levels of resistance were observed under oxic or anoxic conditions (Figure 1; Supplementary Figure S1). These results correlate well with previous general observations showing that arsenite is 100–200 times more toxic than arsenate since arsenite is able to block dithiols groups with high affinity whereas arsenate merely uncouples phosphorylation reactions (Williams and Silver, 1984).

A good number of bacteria have been described for their significant level of resistance to arsenate or arsenite or both (see review in Páez-Espino et al., 2009), e.g., *P. putida* RS-5 [15 mM As(III) and 500 mM As(V)] (Jackson et al., 2005), *Serratia marcescens* [15 mM As(III) and 500 mM As(V)] (Botes et al., 2007) or *Corynebacterium glutamicum* ATCC 13032 [10 mM As(III) and 300 mM As(V)] (Ordóñez et al., 2005). However, other strains are able to tolerate similar or lower concentrations of arsenic than that observed in the CIB strain, e.g., *Stenotrophomonas maltophilia* SA Ant 15 [20 mM As(V)] (Botes et al., 2007) or a β-proteobacterium isolate [100 mM As(V)] (Jackson et al., 2005). The high level of resistance is mainly based in the presence of several copies of the gene clusters involved in arsenic resistance (Andres and Bertin, 2016). The analysis of the *Azoarcus* sp. CIB genome sequence revealed the presence of five genes, *arsRCDAB* (Supplementary Figure S2), that show high sequence identity with those that integrate the *ars* operon for arsenic resistance in many microorganisms (Stolz et al., 2006).

**Figure 2:**
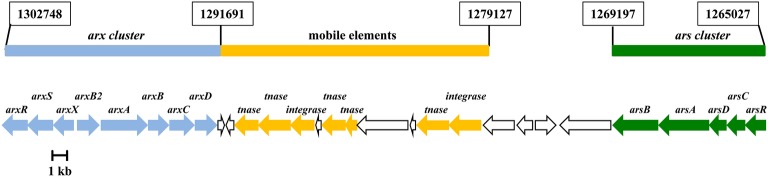
Genetic organization of the *ars* (AzCIB_1124 to AzCIB_1128) and *arx* (AzCIB_1143 to AzCIB_1151) clusters in the arsenic island (genome island V) of *Azoarcus* sp. CIB genome. Genes are represented by arrows. The genes that constitute the *arx* cluster are in blue; genes involved in genetic arrangement and transposition are in yellow (tnase, transposase); genes that constitute the *ars* cluster are in green. Genes of unknown function are indicated in white. Coordinates in the chromosome indicating the start and end of each element (*ars* and *arx* clusters and mobiles elements) are boxed.

The *ars* operon in bacteria is controlled by the ArsR transcriptional regulator (Busenlehner et al., 2003). Three different types of ArsR-like regulators have been identified so far based on the arrangement and location of the three Cys residues that bind the effector molecule arsenite (Wu and Rosen, 1993; Qin et al., 2007; Ordóñez et al., 2008; Murphy and Saltikov, 2009). The first gene of the *ars* cluster of *Azoarcus* sp. CIB encodes the putative ArsR regulator (Figure 2). Comparison of the ArsR sequence from strain CIB with that of selected ArsR regulators of Type 1 (*Escherichia coli* and *Pseudomonas putida*), Type 2 (*Leuconostoc ferriphilum*, *A. ferridurans*, and *Comamonas testosteroni*), and Type 3 (*Corynebacterium glutamicum*) showed that the cysteines that most probably bind to As(III) are located at the C-terminus of the protein (Cys91, Cys92 and Cys99) (Supplementary Figure S3A). A phylogenetic analysis showed that ArsR_CIB_ clusters within the same branch than the Type 2 ArsR regulators analyzed, suggesting a common phylogenetic origin (Supplementary Figure S3B).

The second gene of the *ars* cluster, *arsC*, codes for a putative arsenate reductase that catalyzes the reduction of arsenate to arsenite. Two different families of bacterial detoxifying arsenate reductases, i.e., the glutathione-dependent and the thioredoxin-dependent enzymes, have been recognized (Mukhopadhyay et al., 2002). The arsenate reductase from *Azoarcus* sp. CIB (ArsC_CIB_) clusters within the reductases of the thioredoxin-dependent family, such as the ArsC proteins from *P. putida* (49.3% identity), *Bacillus subtilis* (33% identity) or *Staphylococcus aureus* (30.7% identity), showing the three conserved catalytic Cys residues, i.e., Cys7, Cys80, and Cys88 (Supplementary Figure S4). Interestingly, the genome of the strain CIB harbors another gene annotated as a putative arsenic resistance protein (AzCIB_3861) that shares 62% amino acid sequence identity with the *E. coli* arsenate reductase ArsC but only 21.5% identity with ArsC_CIB_. This gene is surrounded by open reading frames (ORFs) that are not related with arsenic resistance and is located far from the *ars* operon in the genome of strain CIB. The alignment of AzCIB_3861, hereafter named as ArsC2, with other arsenate reductases showed that it clusters within the glutaredoxin family of arsenate reductases (Supplementary Figure S4), suggesting that the evolutionary event of acquisition of *arsC2* might be different than that of the *ars* operon in strain CIB.

The *arsA* and *arsB* genes encode the putative ATPase subunit and the integral membrane protein, respectively, of the ArsAB arsenite-translocating pump that extrudes arsenite (Rosen, 1999, 2002). The ArsA protein has two homologous halves, A1 and A2, connected by a linker of 25 residues. Each half has a consensus nucleotide-binding domain (GKGGVGKTT/S) and As(III)-binding site (DTAPTGH) (Rosen, 1999). The nucleotide and metalloid binding sites are perfectly conserved in ArsA_CIB_ protein (Supplementary Figure S5). The *ars* operon of *Azoarcus* sp. CIB also contains the *arsD* gene that is present only is some of the bacterial *ars* operons described (Yang et al., 2011). The *arsD* gene codes for the arsenite-metallochaperone of the ArsAB pump (Lin et al., 2007). The ArsD protein interacts with ArsA with high affinity when metalloid is bound increasing the efficiency of the ArsAB pump to extrude As(III), hence providing a competitive advantage for growth in environments with moderate amounts of arsenic (Lin et al., 2007). It has been reported that ArsD is an homodimer protein with conserved cysteine residues that form three metalloid binding sites (MBS1-3) (Lin et al., 2007). The cysteines located in ArsD_CIB_ at positions Cys12-Cys13/Cys18, Cys114-Cys115, and Cys122-123 may constitute the MBS1, MBS2, and MBS3 sites (Supplementary Figure S6). It has been described that *ars* operons, harboring *arsDA* genes confer a high level of arsenic resistance (Bhattarcharjee and Rosen, 2007), which might correlate well with the high tolerance of CIB to arsenate (Figure 1; Supplementary Figure S1).

Although some arsenite oxidizing bacteria, e.g., *Azoarcus* sp. DAO1 (Rhine et al., 2006), *Azoarcus* sp. EC-pb1, and *Azoarcus* sp. EC3-pb3 (Sun et al., 2009) have been described within the *Azoarcus* genus, there is no information on the genes involved in arsenic resistance in this bacterial genus. Thus, we used the *Azoarcus* sp. CIB *ars* genes to search for orthologous genes in the so far reported *Azoarcus* genomes. Sequence comparison analyses revealed that the *ars* genes are present in all sequenced *Azoarcus* genomes, and they are arranged in the same orientation in almost all *ars* operons with the exception of *Azoarcus* sp. BH72 and *Azoarcus olearius* DQS-4, which show two copies of *arsC* (*arsC* and *arsC1*) flanking *arsR*, and the *arsDA* genes are located at the end of the operon downstream of *arsB* (Supplementary Figure S1). *Azoarcus* sp. SY36 possesses a unique *ars* organization. The arsenic resistance genes of *Azoarcus* sp. SY36 are arranged in three different operons at different genome locations: (1) *arsRC1B1*, (2) *arsC2H1*, and (3) *arsH2C3B2*. These operons lack the *arsDA* genes, which might explain the increasing number of copies of arsenate reductases (three *arsC*) and arsenite transporters (two copies of *arsB*) to counteract the absence of the arsenite efflux ATPase. In addition, *Azoarcu*s sp. SY36 is the only known *Azoarcus* strain that contains two copies of *arsH*, a gene that encodes a putative methylarsenite oxidase (Yang and Rosen, 2016). All the *arsC* genes present in the *ars* operons of the *Azoarcus* spp. strains sequenced so far encode putative thioredoxin-dependent ArsC proteins suggesting that they share a common evolutionary origin (Supplementary Figure S7). As it was shown in *Azoarcus* sp. CIB, other *Azoarcus* strains also have putative accessory arsenate reductases, e.g., BH72 carries three *arsC* genes (Azo_0525, Azo_1658, and Azo_2067) found outside of the *ars* operon, that belong to the glutaredoxin family and have low percentage of amino acid sequence identity with the thioredoxin-dependent ArsC coded by the *arsC* gene integrated into the *ars* operon. Thus, the presence of accessory *arsC* genes is a relatively common fact that has not been deeply studied yet, and it might reflect a possible differential expression of each *arsC* copy in response to a particular environmental condition, or to other metals/metalloids different to arsenic oxyanions (Páez-Espino et al., 2015). It has been hypothesized that the extra copies of arsenic resistance genes is a general strategy to confer greater tolerance to arsenic in bacteria (Ordóñez et al., 2005; Kang et al., 2015). Regarding the regulation of the *ars* gene expression in the *Azoarcus* genus, the existence of a Type 2 ArsR transcriptional regulator appears to be a common trend in all *ars* operons sequenced so far (Supplementary Figure S8).

### The *ars* Genes Are Involved in Arsenic Resistance in *Azoarcus* sp. CIB

To establish whether the *ars* operon and the *arsC2* gene were involved in the arsenic resistance, we constructed *arsC* (*Azoarcus* sp. CIBd*arsC*), *arsB* (*Azoarcus* sp. CIBd*arsB*), and *arsC2* (*Azoarcus* sp. CIBd*arsC2*) knockout mutant strains (Table 1). All the mutant strains had similar growth rate than that observed at the wild type strains but none of the mutants was able to grow above 75 mM arsenate, which means arsenate resistance decreased to 50% of that of the wild-type strain (Figure 1; Supplementary Figures S9–S12). Since the *Azoarcus* sp. CIBd*arsC* and *Azoarcus* sp. CIBd*arsC*2 mutants show a similar decrease of arsenate resistance, a possible synergistic effect of both arsenate reductases can be suggested. The *Azoarcus* sp. CIBd*arsC* mutant strain showed similar level of arsenite resistance than the wild-type strain (Figure 1; Supplementary Figure S9), which is in agreement with the fact that the ArsC enzyme is not involved in arsenite resistance. However, the lower resistance to arsenate and, specially, to arsenite of the *Azoarcus* sp. CIBd*arsB* strain reveals the importance of the extrusion of arsenite for cell survival and highlights the key role of ArsB in both arsenate and arsenite resistance. Nevertheless, since *Azoarcus* sp. CIBd*arsB* is still able to resist 50 mM of arsenate, we cannot rule out the existence of alternative mechanisms of extrusion of arsenate/arsenite out of the cell. In summary, all these results support the idea that the *arsRCDAB* cluster and the *arsC2* gene are involved in arsenate/arsenite resistance in *Azoarcus* sp. CIB.

**Figure 3:**
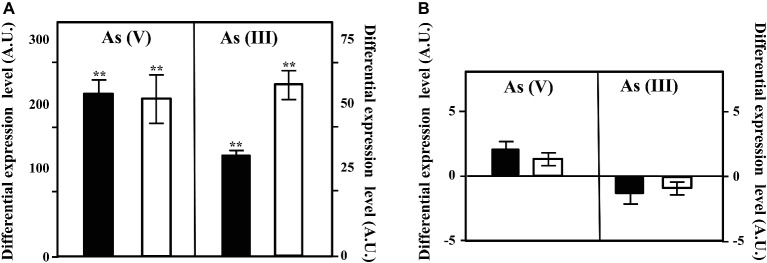
Expression of the *arsC* and *arsC2* genes in *Azoarcus* sp. CIB. Expression of the *arsC* **(A)** or *arsC2* **(B)** genes under aerobic (black columns), or anaerobic (white columns) conditions when the cells were grown until the mid-exponential phase in the presence of 10 mM arsenate [As(V)] or 1 mM arsenite [As(III)]. The differential expression level means the expression of the gene in the presence of arsenic oxyanions with respect to that in the absence of arsenic oxyanions. The mean value and the standard deviation (error bars) of three independent experiments are shown. A.U., arbitrary units. Asterisks indicate that the expression values obtained in the presence of arsenate/arsenite are statistically different based upon Student’s *t* test (^**^p < 0.01).

To check if the cluster *arsRCDAB* and/or the *arsC2* gene are induced in the presence of arsenic, we analyzed the expression of the *arsC* and *arsC2* genes by qRT-PCR in cells cultivated in the presence or absence of arsenic oxyanions. To do that, we extracted total RNA from *Azoarcus* sp. CIB cells grown in pyruvate under aerobic or anaerobic conditions and in the absence or presence of 10 mM arsenate or 1 mM arsenite. The *arsC* gene was shown to be strongly induced in the presence of arsenate and arsenite, both under aerobic and anaerobic conditions (Figure 3A). This result is in agreement with previous reports showing that the *ars* operon is expressed under both aerobic and anaerobic conditions in the presence of arsenic oxyanions in different microorganisms (Saltikov, 2011). In contrast to the clear induction of the *arsC* gene in the presence of arsenate/arsenite, the expression of the *arsC2* gene had not a significant variation in the conditions tested (Figure 3B). This different expression pattern of the two *arsC* genes might reflect a regulatory strategy based on arsenite, rather than arsenate, as inducer molecule of the *ars* operon. As mentioned above, the ArsR transcriptional repressor was shown to bind to the operator region of the *ars* operon and dissociate from the target promoter in the presence of arsenite enabling the transcription of the *ars* genes in many bacteria (Wu and Rosen, 1993; Shi et al., 1994; Zhang et al., 2009; Saltikov, 2011). Hence, the induction of *ars* genes in the presence of arsenate will require some basal expression of an arsenate reductase to generate some arsenite that will trigger a high expression level of the *ars* operon. A minor amount of arsenate reductase within the cell can be achieved either by a basal expression of *arsC* gene (Zhang et al., 2009) or by the presence of additional arsenate reductase encoding genes whose constitutive expression allows the production of arsenite as soon as arsenate reaches the cytoplasm (López-Maury et al., 2009; Fernández et al., 2014). Hence, in *Azoarcus* sp. CIB, the expression of *arsC2* (Figure 3B) might explain the induction of the *ars* genes in the presence of arsenate (Figure 3A). Nevertheless, further work should be performed to unravel the regulatory network that controls the expression of the arsenic resistance genes in *Azoarcus* sp. CIB.

Since *Azoarcus* sp. CIB harbors two genes (*arsC* and *arsC2*) that code for arsenate reductases, both are expressed, and *arsC* is strongly induced by arsenate and is highly probable than the strain CIB holds the ability to reduce arsenate. To confirm this assumption, we collected cells of *Azoarcus* sp. CIB grown aerobically in MC medium supplemented with pyruvate 0.2% after 72 h (end of exponential phase of growth) and we performed a resting cell assay in the presence of arsenate as described in the section “Materials and Methods.” After incubation, arsenite was detected by a modification of a previously described colorimetric protocol (Watanabe et al., 2017). As shown in Supplementary Figure S13, live CIB cells produced the colored arsenite complex from the arsenate added to the medium, whereas boiled cells or 5 mM arsenate added to a culture medium free of cells were not able to produce arsenite. These results strongly suggest that the added arsenate is reduced to arsenite by *Azoarcus* sp. CIB cells, revealing an arsenate reductase activity.

### The *arx* Genes Are Involved in Anaerobic Arsenite Resistance in *Azoarcus* sp. CIB

**Figure 4:**
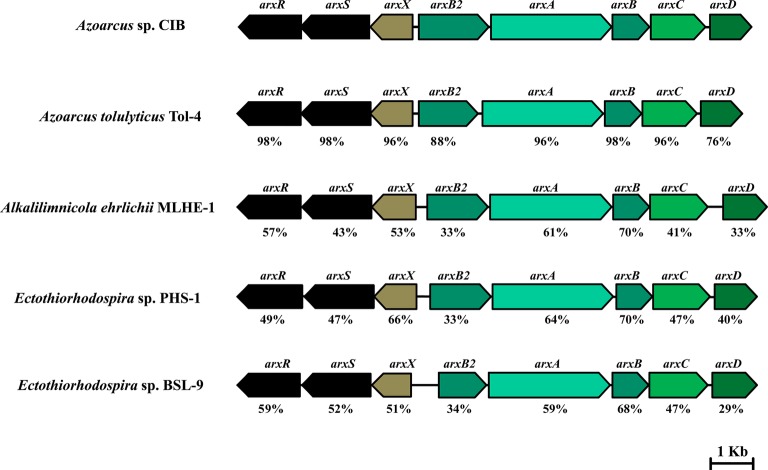
Organization of the *arx* clusters in *Azoarcus* sp. CIB (accession no. NZ_CP011072) (Martín-Moldes et al., 2015), *A. tolulyticus* Tol-4 (accession no. NZ_FTMD00000000), *Alkalilimnicola ehrlichii* MLHE-1 (accession no. NC_008340), *Ectothiorhodospira* sp. PHS-1 (accession no. GCA_000225005.2), and *Ectothiorhodospira* sp. BSL-9 (accession no. NZ_CP011994). Genes are represented by arrows as follows: *arxRS*, regulatory genes; *arxX*, oxyanion binding subunit gene; *arxB2AB*, genes encoding the subunits of the arsenite oxidase; *arxC*, integral electron transport protein gene; and *arxD*, chaperone gene. At the bottom of each gene is indicated its percentage of amino acid sequence identity to the corresponding *Azoarcus* sp. CIB ortholog.

The analysis of the annotated genome sequence of *Azoarcus* sp. CIB indicated the presence of a gene cluster (AzCIB_1143 to AzCIB_1151) (Figure 4) that showed a significant similarity to the *arx* cluster involved in the anaerobic oxidation of arsenite in some bacteria (Oremland et al., 2017). The *arx* cluster has been described mostly in autotrophic bacteria that colonize environments, e.g., alkaline lakes, with elevated concentration of arsenic (Hernández-Maldonado et al., 2017), but also has been reported in facultative oxidizing chemoautotroph such *Alkalimnicola ehrlichii* strain MLHE-1 (Rhine et al., 2006) or anoxygenic photoautrophic anaerobe such as *Ectothiorhodospira* sp. BSL9 (Hernández-Maldonado et al., 2017). However, to our knowledge, it has never been described in a non-extremophile/freshwater bacterium that links the anaerobic As(III) oxidation and nitrate reduction to a metabolism based obligatory on organic compounds. Thus, the presence of *arx* genes in a heterotrophic bacteria, such as *Azoarcus* sp. CIB or the recently sequenced *Azoarcus tolulyticus* Tol-4 strain (Figure 4), represents a new relevant finding.

**Figure 5:**
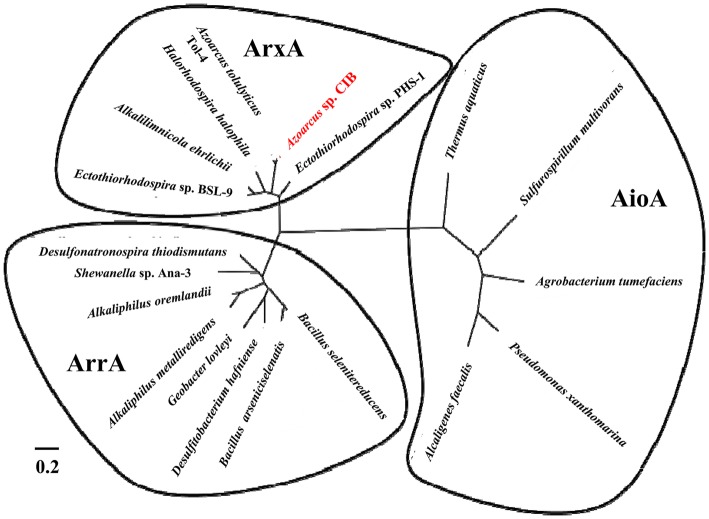
Phylogenetic tree built from the multiple amino acid sequence alignment of the ArxA, ArrA, and AioA proteins. The ArxA proteins belong to *Azoarcus* sp. CIB (WP_050415005), *Ectothiorhodospira* sp. PHS-1 (WP_008932021), *Azoarcus tolulyticus* (WP_076602992), *Halorhodopsira halophila* (WP_011813170), *Alkalilimnicola ehrlichii* (WP_011627967), and *Ectothiorhodospira* sp. BSL-9 (WP_063465591). The ArrA proteins belong to *Desulfonatronospira thiodismutans* (WP_008871035), *Shewanella* sp. ANA-3 (WP_011790217), *Alkaliphilus oremlandii* (WP_012158954), *Alkaliphilus metalliredigens* (ZP_00800578), *Geobacter lovleyi* (WP_012469220), *Desulfitobacterium hafniense* (WP_041272623), *Bacillus arseniciselenatis* (WP_071313526), and *Bacillus selenatireducens* (WP_013173528). The AioA proteins belong to *Thermus aquaticus* (WP_053768293), *Sulfurospirillum multivorans* (WP_025346171), *Agrobacterium tumefaciens* (ABB51928), *Pseudomonas xanthomarina* (WP_041015811), and *Alcaligenes faecalis* (AAQ19838). The clustering to the anaerobic arsenite oxidase (ArxA), respiratory arsenate reductase (ArrA) or aerobic arsenite oxidase (AioA) is indicated.

The *arx* cluster from *Azoarcus* sp. CIB has eight genes organized in two potential divergent operons: (1) *arxXSR*, which codes for a putative arsenite-binding protein (*arxX*) and a two-component regulatory system (*arxSR*), and (2) *arxB2ABCD*, which codes for the putative small subunit (*arxB* and *arxB2*) and large subunit (*arxA*) of the arsenite oxidase, a transmembrane protein involved in electron transfer (*arxC*) and a cytoplasmic chaperone (*arxD*). The genetic organization and the amino acid sequence of the encoded proteins are highly conserved in all *arx* clusters identify so far (Figure 4). The anaerobic arsenite oxidase (code by the arxA gene) is a molybdopterin reductase that belong to the family of the DMSO reductases. It has been described that there is a closest phylogenetic relationship between the arsenite oxidase catalytic subunits (ArxA) and the respiratory arsenate reductases (ArrA) than between the anaerobic (ArxA) and aerobic (AioA) arsenite reductases (Figure 5; Zargar et al., 2010).

**Figure 6:**
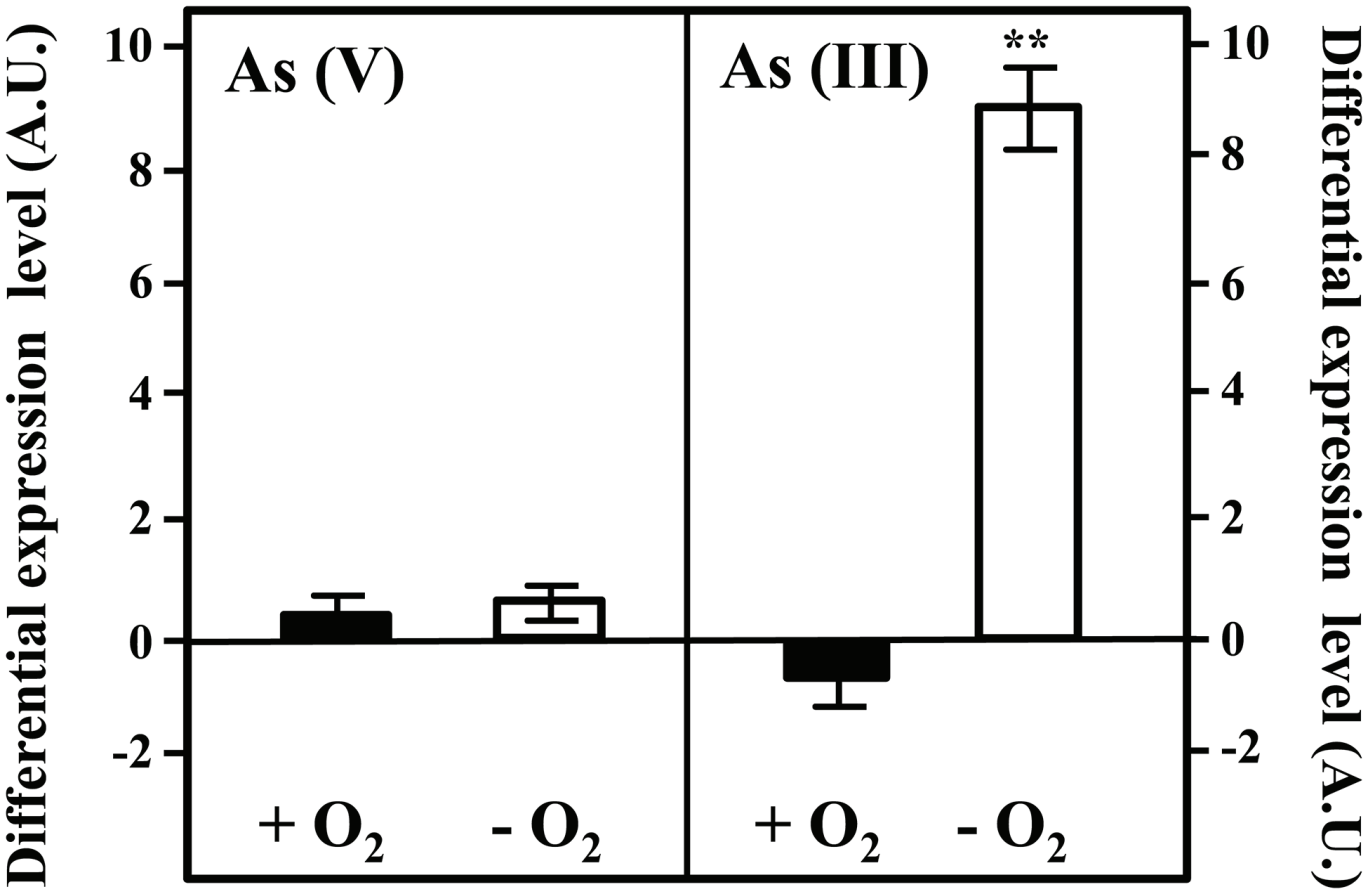
Expression of the *arxA* gene in *Azoarcus* sp. CIB. Expression of the *arxA* gene under aerobic (black columns; +O_2_), or anaerobic (white columns, −O_2_) conditions when the cells were grown until the mid-exponential phase in the presence of 10 mM arsenate [As(V)] or 1 mM arsenite [As(III)]. The differential expression level means the expression of the gene in the presence of arsenic oxyanions with respect to that in the absence of arsenic oxyanions. The mean value and the standard deviation (error bars) of three independent experiments are shown. A.U., arbitrary units. Asterisks indicate that the expression values obtained in the presence of arsenate/arsenite are statistically different based upon Student’s *t* test (^**^p < 0.01).

To check if the gene *arxA*, included in the cluster *arxABCD*, that encode the arsenite oxidase is indeed induced in the presence of arsenic, we analyzed the expression of the *arxA* gene by qRT-PCR in cells cultivated in the presence or absence of arsenic oxyanions. Total RNA was extracted from *Azoarcus* sp. CIB cells grown in pyruvate under aerobic or anaerobic conditions and in the absence or presence of 10 mM arsenate or 1 mM arsenite. The expression of the *arxA* did not significantly change in the presence of arsenate (Figure 6) but increased almost one order of magnitude when arsenite was added to the growth in anaerobic conditions (Figure 6). Interestingly, the expression of *arxA* did not increase; in fact, it had a slight decrease (1.5 times), in aerobic conditions, indicating that anaerobiosis and arsenite, are both needed for the expression of the *arxA* gene. These results are in agreement with previous analysis reported in *A. ehrlichi* MLHE-1 where it has been demonstrated that the *arxA* gene is only induced in the presence of arsenite in anaerobic conditions (Zargar et al., 2010). The anaerobic induction of *arxA* in the presence of arsenite was also observed in *Ectothiorhodospira* PHS-1 where raised up to 140 times (Zargar et al., 2012). The genes *arxRS* encode code the putative response regulator ArxR and the putative sensor kinase ArxS although their role controlling the *arx* cluster has not been experimentally demonstrated yet. However, it has been reported that the products of the genes *aioRS* control the *aio* cluster, responsible for the aerobic oxidation of arsenite in some bacteria (Kashyap et al., 2006; Zargar et al., 2010, 2012; Hernández-Maldonado et al., 2017; Oremland et al., 2017). The expression of the *aio* cluster is inducible by arsenite and by an unknown mechanism of quorum sensing (Kashyap et al., 2006). It is assumed that the regulator AioR does not interact directly with arsenite, is one component of the signal transduction that involves AioX that binds arsenite at periplasm, and the histidine-kinase AioS that transfer the information from the periplasm to the cytoplasm (Kashyap et al., 2006; Saltikov, 2011). It is possible that this scheme is also present in CIB strain with the component of the *arx* cluster, being ArxX protein the periplasmic arsenite binding protein (Badilla et al., 2018), ArxS the sensor kinase responsible for the information transduction to the cytoplasm, and ArxR the response regulator that sense the signal transferred by ArxS. Further experiments about this subject are required to establish the complete network of regulation of the *arx* genes in *Azoarcus* sp. CIB.

**Figure 7:**
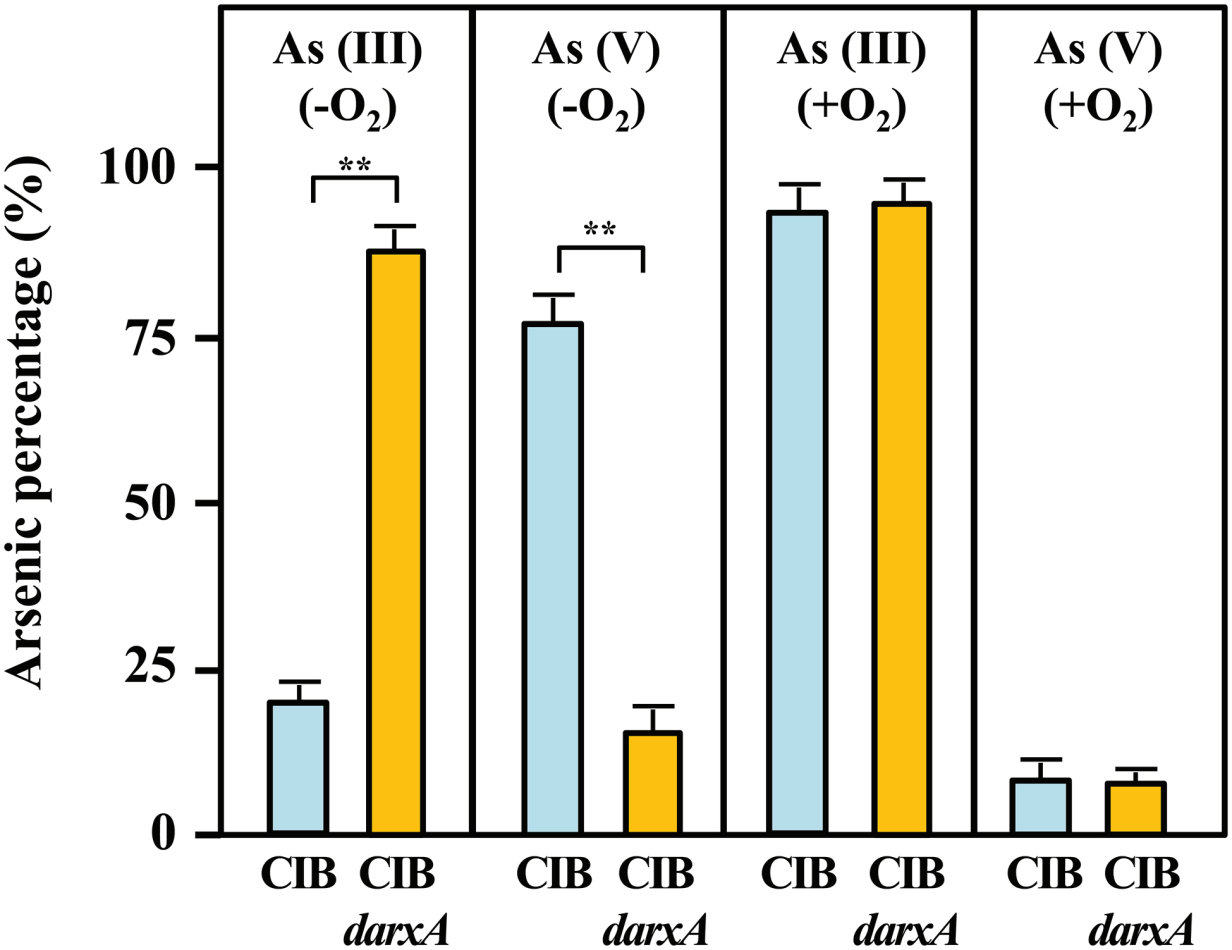
Arsenite oxidation and arsenate production (in percentage) by *Azoarcus* sp. CIB (blue columns) or *Azoarcus* sp. CIBd*arxA* (orange columns) extracts from cells grown 72 h (end of the exponential phase) under anaerobic (−O_2_) or aerobic (+O_2_) conditions in the presence of 0.2 mM arsenite. The arsenic species, arsenite [As(III)] and arsenate [As(V)], were detected by HPLC-ICP as detailed in the section “Materials and Methods,” and the mean of three independent experiments is presented here in percentage. Asterisks indicate the statistical relevance of the As(III) oxidized or As(V) produced based upon Student’s *t* test (^**^p < 0.01).

To experimentally demonstrate that *Azoarcus* sp. CIB has arsenite oxidase activity, we grew cells, under aerobic or anaerobic conditions, in minimal medium with pyruvate as carbon source and in the presence of 0.2 mM arsenite until the end of the exponential phase (around 72 h). The oxidation of arsenite and the production of arsenate was monitored by high performance liquid chromatography-mass spectrometry (HPLC-MS). Whereas under aerobic conditions no significant quantities of arsenate were detected in cell extracts, under anaerobic conditions most of the arsenite (around 80%) was converted into arsenate (Figure 7). Therefore, these results indicate the existence of an arsenite oxidase activity when the CIB strain grows under anoxic conditions.

To ascribe the arsenite oxidase activity observed to the *arx* genes, we constructed an *Azoarcus* sp. CIBd*arxA* mutant strain that harbors a disruptional inactivation of the *arxA* gene (Table 1). Interestingly, the mutant strain was unable to oxidize arsenite either in the presence or absence of oxygen (Figure 7), strongly suggesting that *arxA* encodes the anaerobic arsenite oxidase activity of *Azoarcus* sp. CIB. In addition, the mutant strain *Azoarcus* sp. CIBd*arxA* had a 3-fold decreased level of resistance to arsenite under anaerobic conditions (Figure 1; Supplementary Figure S12). As expected, no decrease in the aerobic resistance to arsenite was observed when the mutant *arxA* strain was compared to the wild-type CIB strain (Figure 1; Supplementary Figure S12). All these results strongly suggest that the *arx* genes encode the enzyme for the anaerobic oxidation of arsenite which enhances the anaerobic resistance of strain CIB to this toxic oxyanion.

### The *arx* Genes Are Involved in the Use of Arsenite as an Extra Energy Source in *Azoarcus* sp. CIB

**Figure 8:**
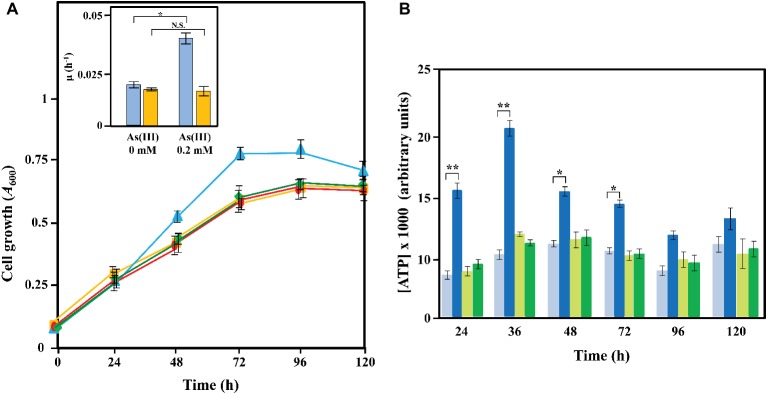
Growth and ATP production of *Azoarcus* sp. CIB strains in the presence or absence of arsenite. **(A)** Time course of the anaerobic growth of *Azoarcus* sp. CIB in the presence (blue line) or absence (yellow line) of 0.2 mM arsenite, and *Azoarcus* sp. CIB*darxA* in the presence (green line) or absence (red line) of 0.2 mM arsenite. The growth in MC medium containing 0.1% pyruvate as carbon source was determined measuring the *A*_600_. In the inset, the growth rates (μ) for *Azoarcus* sp. CIB (blue) and *Azoarcus* sp. d*arxA* (yellow) in the presence (0.2 mM) or absence (0 mM) of arsenite are represented. **(B)** Measurement of the ATP production (as detailed in the section “Materials and Methods”) along the anaerobic growth curves of panel **(A)** of *Azoarcus* sp. CIB in the presence (dark blue) or absence (pale blue) of 0.2 mM arsenite and *Azoarcus* sp. CIBd*arxA* in the presence (dark green) or absence (pale green) of 0.2 mM arsenite. In panels **(A)** and **(B)**, values are the average of three independent experiments. Error bars indicate standard deviations of three independent experiments, and differences were analyzed by Student’s *t* test. N.S., not significant differences (*p* > 0.05); asterisk indicates significant differences (^*^p < 0.05; ^**^p < 0.01).

The oxidation of arsenite to arsenate is a thermodynamically exergonic reaction that can provide energy to support cell growth (Green, 1918; Ehrlich, 2002). In fact, several autotrophic and anoxygenic photosynthetic microorganisms can use arsenite through the activity of anaerobic arsenite oxidase as an extra source of electrons than can be channeled to the respiratory transport chain and contribute to the global energetic status of the cell (Rhine et al., 2005, 2006; Hernández-Maldonado et al., 2017). The *Azoarcus* sp. DAO1 was the first reported *Azoarcus* that can derive energy for growth from metal oxidation coupled to CO_2_ fixation (Rhine et al., 2006). All these strains harbor the RuBisCO genes (Rhine et al., 2006). Although the genome of *Azoarcus* sp. CIB did not reveal the existence of RuBisCO genes (Calvin cycle) or any other genes (e.g., reverse Krebs cycle) likely involved in an autotrophic metabolism, we decided to investigate whether the electrons obtained after the anaerobic oxidation of arsenite by the Arx enzyme might be used as an extra energy source for the cell that could enhance bacterial growth. To do that, we firstly analyzed the growth curves of *Azoarcus* sp. CIB cells grown anaerobically with a limited amount of carbon source (0.1% pyruvate) in the absence or presence of a non-toxic concentration of arsenite. As shown in the inset of Figure 8A, we could observe a significant increase of growth when 0.2 mM arsenite was present. We also tested if it was possible to correlate the enhancement of growth with increasing amounts of arsenite in the medium. Supplementary Figure S14A shows that CIB anaerobically grown with 0.5 mM arsenite was able to reach only slightly higher growth and at 1 mM arsenite this effect disappeared suggesting that CIB might to be more sensitive to arsenite when limiting the amounts of carbon source in the medium. Interestingly, this arsenite-dependent growth increase was not observed with the *Azoarcus* sp. CIBd*arxA* mutant strain (Figure 8A). Hence, these results indicate that arsenite is enhancing growth of strain CIB in an Arx-dependent manner.

To investigate further this observation, we analyzed the ATP production in the anaerobically grown *Azoarcus* sp. CIB and *Azoarcus* sp.CIBd*arxA* cells. As shown in Figure 8B, the addition of arsenite significantly increased, especially during the exponential phase of growth, the amount of ATP produced in *Azoarcus* sp. CIB cells. However, the *Azoarcus* sp.CIBd*arxA* mutant strain was unable to produce an extra amount of ATP in the presence of arsenite (Figure 8B). Furthermore, CIB cells anaerobically grown in 0.5 mM arsenite increased the ATP production more than those cells grown on 0.2 mM arsenite (Supplementary Figure S14). However, this increase in ATP production did not enhance significantly the growth rate suggesting that this extra energy might be used in cellular fitness or another functions not involved in biomass gain. In summary, all these results taken together strongly suggest that the *arx* genes confer to *Azoarcus* sp. CIB the ability to use arsenite as an electron donor that provides an extra energy source for enhancing anaerobic cell growth. As far as we know, this is the first report on the anaerobic utilization of arsenite as an energizing agent in a chemoheterotrophic metabolism.

## Conclusions

In this work, we have identified the genetic determinants involved in arsenic resistance in a member of the *Azoarcus* genus. The *ars* cluster of *Azoarcus* sp. CIB, which encodes the cytoplasmic arsenate reductase and the arsenite exporter, shows significant similarities to other previously characterized bacterial *ars* clusters, and it was used to identify orthologous *ars* genes within the *Azoarcus* genomes so far described (Supplementary Figure S1). Whereas arsenic resistance is widely extended in bacteria, the ability to use arsenite as energy source has been mainly related to extremophile autotrophic bacteria that reside in environments highly contaminated with arsenic oxyanions. In fact, arsenite oxidation in heterotrophic bacteria has been assumed as to be only a detoxification process (Silver and Phung, 2005). However, here we present the first example of an obligate heterotrophic bacterium that obtains extra electrons to enhance anaerobic growth from arsenite oxidation through the expression of the *arx* cluster. Interestingly, the *arx* genes from *Azoarcus* sp. CIB show a GC content of 67.1%, slightly higher than that observed for the CIB genome sequence (65.8%). In addition, the *arx* cluster is separated from the *ars* cluster by putative integrase- and transposase-encoding genes that are located within a chromosomal region that corresponds to genome island V (50 kb) (Figure 2; Martín-Moldes et al., 2015). All these features suggest that the *arx* genes have been acquired by horizontal gene transfer to build an arsenic resistance island in the genome of *Azoarcus* sp. CIB. The arsenic island of strain CIB also supports the notion that metabolic and energetic skills can be gained by genetic mobile elements. The acquisition of mechanisms that provide an extra-energy benefit, such as the arsenite oxidase enzyme, may represent an evolutionary advantage, even for organic degrader bacteria, when the cells drive in environments with low availability of energy sources.

## Data Availability

The raw data supporting the conclusions of this manuscript will be made available by the authors, without undue reservation, to any qualified researcher.

## Author Contributions

GD-R, HF-L, EA-F, MF-M, and MC performed the experiments. GD-R, ED, RM-O, and MC designed the experiments, contributed to the discussion and interpretation of the data. MC and ED wrote the article.

### Conflict of Interest Statement

The authors declare that the research was conducted in the absence of any commercial or financial relationship that could be construed as potential conflict of interest.

## References

[ref1] Anderson G. L. Williams J. Hille R. (1992). The purification and characterization of arsenite oxidase from *Alcaligenes faecalis*, a molybdenum-containing hydroxylase. J. Biol. Chem. 267, 23674–23682. 1331097

[ref2] Andres J. Bertin P. N. (2016). The microbial genomics of arsenic. FEMS Microbiol. Rev. 40, 299–322. 10.1093/femsre/fuv050, PMID: 26790947

[ref3] Badilla C. Osborne T. H. Cole A. Watson C. Djordjevic S. Santini J. M. (2018). A new family of periplasmic-binding proteins that sense arsenic oxyanions. Sci. Rep. 8:6182. 10.1038/s41598-018-24591-w 29674678 PMC5908839

[ref4] Bhattarcharjee H. Rosen B. P. (2007). “Arsenic metabolism in prokaryotic and eukaryotic microbes” in Molecular microbiology of heavy metals. eds. Nies D. H. Silver S. (Heidelberg, Germany: Springer), 371–406.

[ref5] Blázquez B. Carmona M. Díaz E. (2018). Transcriptional regulation of the peripheral pathway for the anaerobic catabolism of toluene and *m*-xylene in *Azoarcus* sp. CIB. Front. Microbiol. 9:506. 10.3389/fmicb.2018.00506, PMID: 29623071 PMC5874301

[ref6] Botes E. van Heerden E. Litthauer D. (2007). Hyper-resistance to arsenic to arsenic in bacteria isolated from an antimony mine in South Africa. S. Afr. J. Sci. 103, 279–281. ISSN: 1996-7489. http://www.scielo.org.za/scielo.php?script=sci_arttext&pid=S0038-23532007000400008&lng=en

[ref7] Busenlehner L. S. Pennella M. A. Giedroc D. P. (2003). The SmtB/ArsR family of metalloregulatory transcriptional repressors: structural insights into prokaryotic metal resistance. FEMS Microbiol. Rev. 27, 131–143. 10.1016/S0168-6445(03)00054-8, PMID: 12829264

[ref8] Cai J. Salmon K. DuBow M. S. (1998). A chromosomal *ars* operon homologue of *Pseudomonas aeruginosa* confers increased resistance to arsenic and antimony in *Escherichia coli*. Microbiology 144, 2705–2713. 9802012 10.1099/00221287-144-10-2705

[ref9] Carmona M. Zamarro M. T. Blázquez B. Durante-Rodríguez G. Juárez J. F. Valderrama J. A. (2009). Anaerobic catabolism of aromatic compounds: a genetic and genomic view. Microbiol. Mol. Biol. Rev. 73, 71–133. 10.1128/MMBR.00021-08 19258534 PMC2650882

[ref10] De Lorenzo V. Timmis K. N. (1994). Analysis and construction of stable phenotypes in gram-negative bacteria with Tn*5*- and Tn*10*-derived minitransposons. Methods Enzymol. 235, 386–405. 8057911 10.1016/0076-6879(94)35157-0

[ref11] Ehrlich H. L. (2002). “Bacterial oxidation of as(III) compounds” in Environmental chemistry of arsenic. ed. Frankenberger W. T. (New York, N.Y: Marcel Dekker, Inc), 313–328.

[ref12] Felsenstein J. (1993). PHYLIP (phylogenetic inference package) version 3.5.1. Seattle, W.A: University of Washington.

[ref13] Fernández M. Udaondo Z. Niqui J. L. Duque E. Ramos J. L. (2014). Synergic role of the two ars operons in arsenic tolerance in *Pseudomonas putida* KT2440. Environ. Microbiol. Rep. 6, 483–489. 10.1111/1758-2229.12167, PMID: 25646541

[ref14] Fernández-Llamosas H. Castro L. Blázquez M. L. Díaz E. Carmona M. (2016). Biosynthesis of selenium nanoparticles by *Azoarcus* sp. CIB. Microb. Cell Factories 15:109. 10.1186/s12934-016-0510-y PMC4908764 27301452

[ref15] Fernández-Llamosas H. Prandoni N. Fernández-Pascual M. Fajardo S. Morcillo C. Díaz E. (2014). *Azoarcus* sp. CIB, an anaerobic biodegrader of aromatic compounds shows an endophytic lifestyle. PLoS One 9:e110771. 10.1371/journal.pone.0110771 25340341 PMC4207700

[ref16] Green H. H. (1918). Description of a bacterium which oxidizes arsenite to arsenate and of one which reduces arsenate to arsenite, isolation from a cattle-dipping tank. S. Afr. Sci. 14, 465–467.

[ref17] Hernández-Maldonado J. Sanchez-Sedillo B. Stoneburner B. Boren A. Miller L. McCann S. (2017). The genetic basis of anoxygenic photosynthetic arsenite oxidation. Environ. Microbiol. 19, 130–141. 10.1111/1462-2920.13509 27555453 PMC5967609

[ref18] Jackson C. R. Horrison K. G. Dugas S. L. (2005). Enumeration and characterization of culturable arsenate resistant bacteria in large estuary. Syst. Appl. Microbiol. 28, 727–734. 10.1016/j.syapm.2005.05.012 16261862

[ref19] Juárez J. F. Zamarro M. T. Eberlein C. Boll M. Carmona M. Díaz E. (2013). Characterization of the *mbd* cluster encoding the anaerobic 3-methylbenzoyl-CoA central pathway. Environ. Microbiol. 15, 148–166. 10.1111/j.1462-2920.2012.02818.x 22759228

[ref20] Kang Y. S. Shi Z. Bothner B. Wang G. McDermott T. R. (2015). Involvement of the Acr3 and DctA anti-porters in arsenite oxidation in *Agrobacterium tumefaciens* 5A. Environ. Microbiol. 17, 1950–1962. 10.1111/1462-2920.12468, PMID: 24674103

[ref21] Kashyap D. R. Botero L. M. Franck W. L. Hassett D. J. McDermott T. R. (2006). Complex regulation of arsenite oxidation in *Agrobacterium tumefaciens*. J. Bacteriol. 188, 1081–1088. 10.1128/JB.188.3.1081-1088.2006 16428412 PMC1347330

[ref22] Kimura M. (1980). A simple method for estimating evolutionary rates of base substitutions through comparative studies of nucleotide sequences. J. Mol. Evol. 16, 111–120. 10.1007/BF01731581, PMID: 7463489

[ref23] Lin Y.-F. Yang J. Rosen B. P. (2007). ArsD: an As(III) metallochaperone for the ArsB As(III)-translocating ATPase. J. Bionerg. Biomembr. 39, 453–458. 10.1007/s10863-007-9113-y 17955352

[ref24] Livak K. J. Schmittgen T. D. (2001). Analysis of relative gene expression data using real-time quantitative PCR and the 2-ΔΔCT. Methods 25, 402–408. 10.1006/meth.2001.1262 11846609

[ref25] López-Barragán M. J. Carmona M. Zamarro M. T. Thiele M. Boll M. Fuchs G. (2004). The *bzd* gene cluster, coding for anaerobic benzoate catabolism in *Azoarcus* sp. CIB. J. Bacteriol. 186, 5462–5774. 10.1128/JB.186.17.5762-5774.2004 PMC516837 15317781

[ref26] López-Maury L. Sánchez-Riego A. M. Reyes J. C. Florencio F. J. (2009). The glutathione/glutaredoxin system is essential for arsenate reduction in *Synechocystis* sp. strain PCC6803. J. Bacteriol. 191, 3534–3543. 10.1128/JB.01798-08, PMID: 19304854 PMC2681892

[ref27] Lundin A. (2000). Use of firefly luciferase in ATP related assays of biomass, enzymes and metabolites. Methods Enzymol. 305, 346–370. 10.1016/S0076-6879(00)05499-9 10812612

[ref28] Martín-Moldes Z. Zamarro M. T. del Cerro C. Valencia A. Gómez M. J. Arcas A. (2015). Whole-genome analysis of *Azoarcus* sp. strain CIB provides genetic insights to its different lifestyles and predicts novel metabolic features. Syst. Appl. Microbiol. 38, 462–471. 10.1016/j.syapm.2015.07.002 26259823

[ref29] Mead M. N. (2005). Arsenic: in search of an antidote to a global poison. Environ. Health Prospects 113, A378–A386. 10.1289/ehp.113-a378 PMC1257621 15929879

[ref30] Miller J. H. (1972). Experiments in molecular genetics. Cold Spring Harbor, N.Y: Cold Spring Harbor Laboratory Press.

[ref31] Mukhopadhyay R. Rosen B. P. (2002). Arsenate reductases in prokaryotes and eukaryotes. Environ. Health Perspect. 110, 745–748. 10.1289/ehp.02110s5745, PMID: 12426124 PMC1241237

[ref32] Mukhopadhyay R. Rosen B. P. Phung L. T. Silver S. (2002). Microbial arsenic: from geocycles to genes and enzymes. FEMS Microbiol. Rev. 26, 311–325. 10.1111/j.1574-6976.2002.tb00617.x 12165430

[ref33] Murphy J. N. Saltikov C. W. (2009). The ArsR repressor mediates arsenite-dependent regulation of arsenate respiration and detoxification operons of *Shewanella* sp. strain ANA-2. J. Bacteriol. 191, 6722–6731. 10.1128/JB.00801-09 19717602 PMC2795299

[ref34] Oden K. L. Gladysheva T. B. Rosen B. P. (1994). Arsenate reduction mediated by the plasmid-encoded *arsC* protein is coupled to glutathione. Mol. Microbiol. 12, 301–306. 10.1111/j.1365-2958.1994.tb01018.x, PMID: 8057854

[ref35] Ordóñez E. Letek M. Valbuena N. Gil A. J. Mateos L. M. (2005). Analysis of genes involved in arsenic resistance in *Corynebacterium glutamicum* ATCC 13032. Appl. Environ. Microbiol. 71, 6206–6215. 10.1128/AEM.71.10.6206-6215.2005, PMID: 16204540 PMC1266000

[ref36] Ordóñez E. Thiyagarajan S. Cook J. D. Stemmler T. L. Gil J. A. Mateos L. M.. (2008). Evolution of metal(loid) binding sites in transcriptional regulators. J. Biol. Chem. 283, 25706–25714. 10.1074/jbc.M803209200, PMID: 18591244 PMC2533085

[ref37] Oremland R. S. Saltikov C. W. Stolz J. F. Hollibaugh J. T. (2017). Autotrophic microbial arsenotrophy in arsenic-rich soda lakes. FEMS Microbiol. Lett. 364, 443–467. 10.1093/femsle/fnx146 28859313

[ref38] Oremland R. S. Stolz J. F. (2005). Arsenic, microbes and contaminated aquifers. Trends Microbiol. 13, 45–49. 10.1016/j.tim.2004.12.002 15680760

[ref39] Páez-Espino A. D. Durante-Rodríguez G. de Lorenzo V. (2015). Functional coexistence of twin arsenic resistance systems in *Pseudomonas putida* KT2440. Environ. Microbiol. 17, 229–238. 10.1111/1462-2920.12464, PMID: 24673935

[ref40] Páez-Espino D. Tamames J. de Lorenzo V. Cánovas D. (2009). Microbial responses to environmental arsenic. Biometals 22, 112–130. 10.1007/s10534-008-9195-y 19130261

[ref41] Qin J. Fu H. L. Ye J. Bencze K. Z. Stemmler T. L. Rawlings D. E.. (2007). Convergent evolution of a new arsenic binding site in the ArsR/SmtB family of metalloregulators. J. Biol. Chem. 282, 34346–34355. 10.1074/jbc.M706565200, PMID: 17897948 PMC2859433

[ref42] Rhine E. D. Garcia-Dominguez E. Phelps C. D. Young L. Y. (2005). Environmental microbes can speciate and cycle arsenic. Environ. Sci. Technol. 39, 9569–9573. 10.1021/es051047t, PMID: 16475337

[ref43] Rhine E. D. Phelps C. D. Young L. Y. (2006). Anaerobic arsenite oxidation by novel denitrifying isolates. Environ. Microbiol. 8, 899–908. 10.1111/j.1462-2920.2005.00977.x, PMID: 16623746

[ref44] Rosen B. P. (1999). Families of arsenic transporters. Trends Microbiol. 7, 207–212. 10.1016/S0966-842X(99)01494-8, PMID: 10354596

[ref45] Rosen B. P. (2002). Biochemistry of arsenic detoxification. FEBS Lett. 529, 86–92. 10.1016/s0014-5793(02)03186-1 12354618

[ref46] Saitou N. Nei M. (1987). The neighbor-joining method: a new method for reconstructing phylogenetic trees. Mol. Biol. Evol. 4, 406–425. 3447015 10.1093/oxfordjournals.molbev.a040454

[ref47] Saltikov C. W. (2011). “Regulation of arsenic metabolic pathways in prokaryotes” in Microbial metal and metalloid metabolism. eds. Stolz J. Oremlands R. (Washington DC: ASM Press), 195–210.

[ref48] Sambrook J. Rusell D. (2001). Molecular cloning: A laboratory manual. New York: Cold Spring Harbor Laboratory Press.

[ref49] Sanger F. Nicklen S. Coulson A. R. (1977). DNA sequencing with chain-termining inhibitors. Proc. Natl. Acad. Sci. USA 74, 5463–5467. 271968 10.1073/pnas.74.12.5463 PMC431765

[ref50] Schäfer A. Tauch A. Jäger W. Kalinowski J. Thierbach G. Pühler A. (1994). Small mobilizable multi-purpose cloning vectors derived from the *Escherichia coli* pK18 and pK19: selection of defined deletions in the chromosome of *Corynebacterium glutamicum*. Gene 145, 69–73. 8045426 10.1016/0378-1119(94)90324-7

[ref51] Shi W. Wu J. Rosen B. P. (1994). Identification of a putative metal binding site in a new family of metalloregulatory proteins. J. Biol. Chem. 269, 19826–19829. PMID: 8051064

[ref52] Silver S. Phung L. T. (2005). Genes and enzymes involved in bacterial oxidation and reduction of inorganic arsenic. Appl. Environ. Microbiol. 71, 599–608. 10.1128/AEM.71.2.599-608.2005, PMID: 15691908 PMC546828

[ref53] Stolz J. F. Basu P. Oremland R. S. (2010). Microbial arsenic metabolism: new twists on an old poison. Microbe Mag. 5, 53–59. 10.1128/microbe.5.53.1

[ref54] Stolz J. F. Basu P. Santini J. M. Oremland R. S. (2006). Arsenic and selenium in microbial metabolism. Annu. Rev. Microbiol. 60, 107–130. 10.1146/annurev.micro.60.080805.142053 16704340

[ref55] Stolz J. F. Oremland R. S. (1999). Bacterial respiration of arsenic and selenium. FEMS Microbiol. Rev. 23, 615–627. 10.1111/j.1574-6976.1999.tb00416.x, PMID: 10525169

[ref56] Sun W. Sierra-Alvarez R. Fernández N. Sanz J. L. Amils R. Legatzki A.. (2009). Molecular characterization and *in situ* quantification of anoxic arsenite-oxidizing denitrifying enrichment cultures. FEMS Microbiol. Ecol. 68, 72–85. 10.1111/j.1574-6941.2009.00653.x, PMID: 19187211 PMC4532341

[ref57] Thompson J. D. Higgins D. G. Gibson T. J. (1994). CLUSTAL W: improving the sensitivity of progressive multiple sequence alignment through sequence weighting, position-specific gap penalties and weight matrix choice. Nucleic Acids Res. 22, 4673–4680. 10.1093/nar/22.22.4673, PMID: 7984417 PMC308517

[ref58] Turner A. W. (1949). Bacterial oxidation of arsenite. Nature 164, 76–77. 18133543 10.1038/164076a0

[ref59] Valderrama J. A. Durante-Rodríguez G. Blázquez B. García J. L. Carmona M. Díaz E. (2012). Bacterial degradation of benzoate: cross-regulation between aerobic and anaerobic pathways. J. Biol. Chem. 287, 10494–10508. 10.1074/jbc.M111.309005 22303008 PMC3322966

[ref60] Van Lis R. Nitschke W. Duval S. Schoepp-Cothenet B. (2013). Arsenic as bioenergetic substrates. Bioch. Biophysic. Acta 1827, 176–188. 10.1016/j.bbabio.2012.08.007 22982475

[ref61] Watanabe T. Miura A. Iwata T. Kojima H. Fukul M. (2017). Dominance of *Sulfuritalea* species in nitrate-depleted water of a stratified freshwater lake and arsenate respiration ability within the genus. Environ. Microbiol. Rep. 9, 522–527. 10.1111/1758-2229.12557, PMID: 28618172

[ref62] Williams J. W. Silver S. (1984). Bacterial resistance and detoxification of heavy metals. Enzym. Microb. Technol. 6, 530–537. 10.1016/0141-0229(84)90081-4

[ref63] Wu J. Rosen B. P. (1993). Metalloregulated expression of the *ars* operon. J. Biol. Chem. 268, 52–58. PMID: 8416957

[ref64] Yamamura S. Amachi S. (2014). Microbiology of inorganic arsenic: from metabolism to bioremediation. J. Biosci. Bioeng. 118, 1–9. 10.1016/j.jbiosc.2013.12.011 24507904

[ref65] Yang H. C. Rosen B. P. (2016). New mechanisms of bacterial arsenic resistance. Biochem. J. 39, 5–13. 10.1016/j.bj.2015.08.003 PMC6138428 27105594

[ref66] Yang J. Salam A. A. Rosen B. P. (2011). Genetic mapping of the interface between the ArsD metallochaperone and the ArsA ATPase. Mol. Microbiol. 79, 872–881. 10.1111/j.1365-2958.2010.07494.x 21299644 PMC3079357

[ref67] Zargar K. Conrad A. Bernick D. L. Lowe T. M. Stolc V. Hoeft S. (2012). ArxA, a new clade of arsenite oxidase within the DMSO reductase family of molybdenum oxidoreductases. Environ. Microbiol. 14, 1635–1645. 10.1111/j.1462-2920.2012.02722.x 22404962

[ref68] Zargar K. Hoeft S. Oremland R. Saltikov C. W. (2010). Identification of a novel arsenite oxidase gene, *arxA*, in the haloalkaliphilic, arsenite-oxidating bacterium *Alkalilimnicola ehrlichii* strain MLHE-1. J. Bacteriol. 192, 3755–3762. 10.1128/JB.00244-10 20453090 PMC2897359

[ref69] Zhang Y. B. Monchy S. Greenberg B. Mergeay M. Gang O. Taghavi S.. (2009). ArsR arsenic-resistance regulatory protein from *Cupriavidus metallidurans* CH34. Antonie Van Leeuwenhoek 96, 161–170. 10.1007/s10482-009-9313-z, PMID: 19238575

[ref70] Zhu Y. G. Yoshinaga M. Zhao F.-J. Rosen B. P. (2014). Earth abides arsenic biotransformations. Annu. Rev. Earth Planet. Sci. 42, 443–467. 10.1146/annurev-earth-060313-054942 26778863 PMC4712701

