# Supplementary material for: ArxA From Azoarcus sp. CIB, an Anaerobic Arsenite Oxidase From an Obligate Heterotrophic and Mesophilic Bacterium
Source: Front Microbiol. 2019 Jul 30;10:1699. doi: 10.3389/fmicb.2019.01699 (PMC6683785; doi:10.3389/fmicb.2019.01699)
Supplement: Supplementary file 1 [file Data_Sheet_1.pdf]

SUPPLEMENTARY MATERIALS

ArxA from *Azoarcus* sp. CIB, an anaerobic arsenite oxidase from an obligate heterotrophic and mesophilic bacterium

Gonzalo Durante-Rodríguez[§1], Helga Fernández-Llamosas[§1], Elena Alonso-Gonzales[1], María Nieves Fernández-Muñiz[2], Riansares Muñoz-Olivas[2], Eduardo Díaz[1], and Manuel Carmona[1*]

1. Microbial and Plant Biotechnology Department. Centro de Investigaciones Biológicas-CSIC. Ramiro de Maeztu 9, 28040 Madrid, Spain
2. Departamento de Química Analítica, Facultad de Químicas, Complutense University of Madrid, Avenida Complutense s/n, 28040 Madrid, Spain

§ G.D-R and H.F-Ll. contributed equally to this work.
Correspondence: Dr. Manuel Carmona.
mcarmona@cib.csic.es

SUPPLEMENTARY Figure Legends

FIGURE S1. Time course of the growth of *Azoarcus* sp. CIB in aerobic (**A** and **B**) or anaerobic (**C** and **D**) conditions in the presence of increasing concentrations of arsenate (**A** and **C**) or arsenite (**B** and **D**). The means of three independent experiments are represented and error bars show the standard deviation. The color code for each concentration of arsenic oxyanion used is indicated.

FIGURE S2. Organization of the *ars* gene clusters in *Azoarcus* sp. CIB (accession no. NZ_CP011072) (Martín-Moldes *et al*., 2014), *A. tolulyticus* ATCC51758 (accession no. NZ_FTMD00000000), *Azoarcus* sp. KH32C (accession no. NC_020516) (Nishizawa *et al*., 2012), "*Aromatoleum aromaticum*" EbN1 (accession no. NC_006513) (Kube, 2004), *Azoarcus* sp. PA01 (accession no. NZ_LARU01000000) (Junghare *et al*., 2015), *Azoarcus* sp. BH72 (accession no. NC_008702) (Krause *et al*., 2006), *Azoarcus olearius* DQS-4 (accession no. NZ_CP016210.1) (Faoro *et al*., 2017) and *Azoarcus* sp. SY36 (accession no. CP025682). Genes are represented by arrows and they are predicted to encode: black, regulatory protein; orange, arsenate reductase; grey, arsenite metallochaperone; green, arsenite-translocating ATPase; red, arsenite transport protein; yellow, methylarsenite oxidase. At the bottom of each gene is indicated its percentage of amino acid sequence identity to the corresponding *Azoarcus* sp. CIB ortholog. Two vertical lines indicate that the genes are not adjacent in the genome.

FIGURE S3. Multiple sequence alignment of ArsR from *Azoarcus* sp. CIB with ArsR regulators from other bacteria. **(A)** The ArsR protein sequences belong to Type 1 (*Leptospirillum ferriphilum*, AAY85166; *Acidithiobacillus ferridurans*, AAF69241; and *Comamonas testosteroni*, WP_080553873); Type 2 (*E. coli*, AVD50083; *P. putida*, WP_010952951) or Type 3 (*Corynebacterium glutamicum* N, BAQ21168). Pale grey shows identical residues in all ArsR sequences. Green indicates identical residues in more than 80% of ArsR sequences. Cys residues are indicated in yellow and boxed in red are the key Cys residues in Type 2 ArsR regulators. (**B)** Phylogenetic tree built from the multiple amino acid sequence alignment of the ArsR proteins using the program Phylogeny. The clustering of each ArsR into Type 1, Type 2 or Type 3 is indicated.

FIGURE S4. Multiple sequence alignment of ArsC from *Azoarcus* sp. CIB with ArsC proteins from other bacteria. (**A)** The ArsC protein sequences belong to *Escherichia coli* (WP_000065769), *Neisseria gonorrhoeae* (WP_003701800), *Pseudomonas aeruginosa* (WP_003120710), *Yersinia enterocolitica* (WP_005162101), *Staphylococcus aureus* (WP_000358995), *Bacillus subtilis* (EHA31227), *Azoarcus* sp. CIB (WP_050414990, ArsC, CIB1; WP_050417386, ArsC2, CIB2) and *Pseudomonas putida* (CAC18654). Green and yellow indicate amino acid sequence identity in more than 90% of the ArsC sequences compared from the glutaredoxin-dependent (G) and thioredoxin-dependent (T) families, respectively. Boxed are the cysteines and arginines involved in As(III) interaction. **(B)** Phylogenetic tree built from the multiple amino acid sequence alignment of the ArsC proteins. The clustering of each ArsC to the glutaredoxin-dependent (G) or thioredoxin-dependent (T) families is indicated in green and yellow, respectively.

FIGURE S5. Multiple sequence alignment of ArsA from different *Azoarcus* spp. The ArsA protein sequences belong to *Azoarcus* sp. BH72 (WP_011766087), *A. olearius*

sp. DQS-1 (WP_065340640), *Azoarcus* sp. KH32 (WP_015451705), *A. toluclasticus* (WP_018990472), *Azoarcus* sp. CIB (WP_050418239), *A. tolulyticus* (WP_076603133), *Azoarcus* sp. PA01 (WP_053422586) and "*Aromatoleum aromaticum*" EbN1 (WP_011238648). Grey shows identical residues in all ArsD sequences. Yellow and boxed in red shows the conserved cysteines. Green and boxed in blue indicates the ATPase region. Blue and boxed in violet indicates the region of interaction with As(III). Sequences were aligned using multiple sequence alignment program Clustal (Thompson *et al*., 1994). Amino acids are indicated by their standard one-letter-code.

FIGURE S6. Multiple sequence alignment of ArsD from different *Azoarcus* strains. The ArsD protein sequences belong to *Azoarcus* sp. BH72 (WP_011766086), *A. olearius* sp. DQS-1 (WP_065340639), *Azoarcus* sp. KH32 (WP_015451704), *A. toluclasticus* (WP_018990471), *Azoarcus* sp. CIB (WP_050414991), *A. tolulyticus* (WP_076602999), *Azoarcus* sp. PA01 (WP_053422222) and "*Aromatoleum aromaticum*" EbN1 (WP_011238647). Grey shows identical residues in all ArsD sequences. Yellow indicates conserved cysteines in putative MBS1-3 sites, and boxed in red are the cysteines likely involved in As(III) interaction in MBS1. Sequences were aligned using multiple sequence alignment program Clustal (Thompson *et al*., 1994). Amino acids are indicated by their standard one-letter-code.

FIGURE S7. Multiple sequence alignment of ArsC from *Azoarcus* sp. CIB with putative ArsC reductases from other *Azoarcus* strains**.** The ArsC protein sequences belong to *Azoarcus* sp. BH72 (1) (WP_011766082) and (2) (WP_011766084), *Azoarcus* sp. KH32 (WP_015451703), "*Aromatoleum aromaticum*" EbN1 (WP_011238646), *Azoarcus* sp. PA01 (WP_053422223), *Azoarcus* sp. CIB (WP_050414990) and *Azoarcus toluclasticus* (WP_018990470). Grey indicates identical residues in at least 90% of ArsC sequences. Yellow indicates identical residues in more than 80% of ArsC sequences. Yellow boxed in red shows the putative cysteines involved in As(III) interaction. Sequences were aligned using multiple sequence alignment program Clustal (Thompson *et al*., 1994). Amino acids are indicated by their standard one-letter-code.

FIGURE S8. Multiple sequence alignment of ArsR from *Azoarcus* sp. CIB with putative ArsR regulators from other *Azoarcus* strains. The ArsR protein sequences are as follows: QS, *Azoarcus olearius* DQS-4 (WP_065340638); BH72, *Azoarcus* sp. BH72 (WP_011766083); PA01, *Azoarcus* sp. PA01 (WP_053422224); EbN1, "*Aromatoleum aromaticum*" EbN1 (WP_011238645); CIB, *Azoarcus* sp. CIB (WP_050414989); tolul, *Azoarcus tolulyticus* (WP_076603001); tolu, *Azoarcus toluclasticus* (WP_018990469); SY39, *Azoarcus* sp. SY39 (WP_102245597) and KH32C, *Azoarcus* sp. KH32C (WP_015451702). Green shows identical residues in all ArsR sequences. Blue indicates identical residues in more than 80% of ArsR sequences. Yellow boxed in red shows the cysteine residues likely involved in As(III) interaction. Sequences were aligned using multiple sequence alignment program Clustal (Thompson *et al*., 1994). Amino acids are indicated by their standard one-letter-code.

FIGURE S9. Time course of the growth of *Azoarcus* sp. CIBd*arsC* in aerobic (**A** and **B**) or anaerobic conditions (**C** and **D**) in the presence of increasing concentrations of arsenate (**A** and **C**) or arsenite (**B** and **D**). The means of three independent experiments are represented and error bars show the standard deviation. The color code for each concentration of arsenic oxyanion used is indicated.

FIGURE S10. Time course of the growth of *Azoarcus* sp. CIBd*arsB* in aerobic (**A** and **B**) or anaerobic conditions (**C** and **D**) in the presence of increasing concentrations of arsenate (**A** and **C**) or arsenite (**B** and **D**). The means of three independent experiments are represented and error bars show the standard deviation. The color code for each concentration of arsenic oxyanion used is indicated.

FIGURE S11. Time course of the growth of *Azoarcus* sp. CIBd*arsC2* in aerobic (**A** and **B**) or anaerobic conditions (**C** and **D**) in the presence of increasing concentrations of arsenate (**A** and **C**) or arsenite (**B** and **D**). The means of three independent experiments are represented and error bars show the standard deviation. The color code for each concentration of arsenic oxyanion used is indicated.

FIGURE S12. Time course of the growth of *Azoarcus* sp. CIBd*arxA* in aerobic (**A** and **B**) or anaerobic conditions (**C** and **D**) in the presence of increasing concentrations of arsenate (**A** and **C**) or arsenite (**B** and **D**). The means of three independent experiments are represented and error bars show the standard deviation. The color code for each concentration of arsenic oxyanion used is indicated.

FIGURE S13. Colorimetric assay of arsenite production in *Azoarcus* sp. CIB cells cultured in the presence of arsenate. In the left column are shown the filters stained with the colored DEDTC-arsenite complexes formed with increasing concentrations of arsenite. The right column shows the filters stained with the colored DEDTC-arsenite complexes formed by CIB resting cells incubated in the presence of 5 mM arsenate (MC-Pyr CIB), and the controls that show that neither arsenate 5 mM added to the culture medium (MC-Pyr) nor boiled CIB resting cells incubated in the presence of 5 mM arsenate (MC-Pyr Boiled CIB) were able to produce detectable DEDTC-arsenite complexes.

FIGURE S14. Growth and ATP production of *Azoarcus* sp. CIB cells in the presence of increasing concentrations of arsenite. (**A)** Time course of the anaerobic growth of *Azoarcus* sp. CIB in the presence of 0.2 mM (blue line), 0.5 mM (red line), 1 mM (violet line) or absence (yelow line) of arsenite. The growth in MC medium containing 0.1 % pyruvate as carbon source was determined by measuring the A_{600}. (**B**) Measurement of the ATP production (as detailed in Material and Methods) along the anaerobic growth curves of panel **A** of *Azoarcus* sp. CIB in the presence of 0.2 mM (blue columns), 0.5 mM (red columns), 1 mM (violet columns) or absence (yelow columns) of arsenite. Values represent the average of three independent experiments. Error bars indicate standard deviations and differences were analyzed by Student's test. Asterisks indicate significant differences (*, $P<0.05$; **, $P<0.01$).

FIGURE S15. Confirmation by PCR analysis of the constructed *Azoarcus* sp. CIB mutant strains. **A.** Lanes 2 (primers 5'ext-arsC and F24) and 5 (primers 3'ext-arsC and R24) show the fragments of 564 bp and 643 bp, respectively, that correspond to the expected insertion into the *arsC* gene. Lane 1 (primers 5'ext-arsC and 3'ext-arsC) does not show any band indicating that a wild-type *arsC* gene has not been reconstructed after the recombination. Lanes 3 (primers 5'ext-arsC and R24) and 4 (primers 3'ext-arsC and F24) do not show the fragments of 583 bp and 630 bp, respectively, that should be expected if the insertion had been produced in the opposite orientation. Lanes 7 (primers 5'ext-arsC2 and F24) and 10 (primers 3'ext-arsC2 and R24) show he

fragments of 913 bp and 760 bp, respectively, that correspond to the expected insertion into the *arsC2* gene. Lane 6 (primers 5'ext-arsC2 and 3'ext-arsC2) does not show any band indicating that a wild-type *arsC2* gene has not been reconstructed after the recombination. Lanes 8 (primers 5'ext-arsC2 and R24) and 9 (primers 3'ext-arsC2 and F24) do not show the fragments of 932 bp and 741 bp, respectively, that should be expected if the insertion had been produced in the opposite orientation. Controls of genomic DNA isolated from *Azoarcus* sp. CIB wild type strain amplified with primers 5'ext-arsC and 3'ext-arsC (generate a fragment of 620 bp) and with primers 5'ext-arsC2 and 3'ext-arsC2 (generate a fragment of 989 bp) are shown in lanes 11 and 12, respectively. Lanes M, 1 kb DNA ladder from New England BioLabs used as size marker in the electrophoresis. **B.** Lanes 3 (primers 5'ext-arxA and R24) and 4 (primers 3'ext-arxA and F24) show the fragments of 2223 bp and 1268 bp, respectively, that correspond to the expected insertion into the *arxA* gene. Lane 1 (primers 5'ext-arxA and 3'ext-arxA) does not show any band indicating that a wild-type *arxA* gene has not been reconstructed after the recombination. Lanes 2 (primers 5'ext-arxA and F24) and 4 (primers 3'ext-arxA and R24) do not show the fragments of 2203 bp and 1288 bp, respectively, that should be expected if the insertion had been produced in the opposite orientation. Lanes 7 (primers 5'ext-arsB and F24) and 10 (primers 3'ext-arsB and R24) show the fragments of 1299 bp and 1013 bp, respectively, that correspond to the expected insertion into the *arsB* gene. Lane 6 (primers 5'ext-arsB and 3'ext-arsB) does not show any band indicating that the wild-type *arsB* gene has not been reconstructed after the recombination. Lanes 8 (primers 5'ext-arsB and R24) and 9 (primers 3'ext-arsB and F24) do not show the fragments of 1318 bp and 994 bp, respectively, that should be expected if the insertion had been produced in the opposite orientation. Controls of genomic DNA isolated from *Azoarcus* sp. CIB wild type strain amplified with primers 5'ext-arxA and 3'ext-arxA (generate a fragment of 2872 bp) and with primers 5'ext-arsB and 3'ext-arsB (generate a fragment of 1631 bp) are shown in lanes 11 and 12, respectively. Lanes M, 1 kb DNA ladder from New England BioLabs used as size marker in the electrophoresis.

REFERENCES

Faoro, H., Rene Menegazzo, R., Battistoni, F., Gyaneshwar, P., do Amaral, F. P., Taulé, C., *et al*. (2017). The oil-contaminated soil diazotroph *Azoarcus olearius* DQS-4^T is genetically and phenotypically similar to the model grass endophyte *Azoarcus* sp. BH72. *Environ. Microbiol. Rep.* 9, 223-238.

Junghare, M., Patil, Y., and Schink, B. (2015). Draft genome sequence of a nitrate-reducing, *o*-phthalate degrading bacterium, *Azoarcus* sp. strain PA01(T). *Stand. Genomic. Sci.* 10, 90. doi: 10.1186/s40793-015-0079-9

Krause, A., Ramakumar, A., Bartels, D., Battistoni, F., Bekel, T., Boch, J., *et al.* (2006). Complete genome of the mutualistic, N_2-fixing grass endophyte *Azoarcus* sp. strain BH72. *Nat. Biotechnol.* 24, 1385-1391.

Kube, M., Heider, J., Hufnagel, P., Kühner, S., Beck, A., Reinhardt, R., and Rabus, R. (2004), Genes involved in the anaerobic degradation of toluene in a denitrifying bacterium, strain EbN1. *Arch. Microbiol.* 181,182-184.

Nishizawa, T., Tago, K., Oshima, K., Hattori, M., Ishii, S., Otsuka, S., and Senoo, K. (2012). Complete genome sequence of the denitrifying and N_2O-reducing bacterium *Azoarcus* sp. strain KH32C. *J. Bacteriol*. 194: 1255.

Thompson, J. D., Higgins, D. G., and Gibson, T. J. (1994) CLUSTAL W: improving the sensitivity of progressive multiple sequence alignment through sequence weighting, position-specific gap penalties and weight matrix choice. *Nucleic Acids Res.* 22, 4673-4680.

Table S1. *Azoarcus* sp. CIB genes putatively involved in metal/metalloids resistance

Gene ID	Gene name	Genome position	Predicted role
**Arsenic**			
AzCIB_1124	*arsR*	1265027 - 1265398	Transcriptional regulator
AzCIB_1125	*arsC*	1265395 - 1265874	Arsenate reductase
AzCIB_1126	*arsD*	1265919 - 1266278	Arsenite metallochaperone
AzCIB_1127	*arsA*	1266290 - 1268107	Arsenite efflux pump ATPase
AzCIB_1128	*arsB*	1268091 - 1269197	Arsenite efflux pump
AzCIB_1144	*arxD*	1293004 - 1292153	Chaperone
AzCIB_1145	*arxC*	1294217 - 1293093	Integral electron transport protein
AzCIB_1146	*arxB*	1294989 - 1294231	Arsenite oxidase subunit
AzCIB_1147	*arxA*	1297492 - 1295003	Arsenite oxidase subunit
AzCIB_1148	*arxB2*	1298996 - 1297551	Arsenite oxidase subunit
AzCIB_1149	*arxX*	1299088 - 1299951	Arsenite high affinity protein
AzCIB_1150	*arxS*	1299960 - 1301417	TCS sensor regulatory protein
AzCIB_1151	*arxR*	1301414 - 1302748	TCS response regulatory protein
AzCIB_3861	*arsC2*	4312406 - 4312765	Arsenate reductase
**Cobalt, zinc, cadmium**			
AzCIB_0579		0675276 - 0674098	Efflux protein
AzCIB_1623		1831358 - 1830978	Protein conserved in *czc* clusters
AzCIB_1624	*czcA3*	1834528 - 1831409	CzcA outer membrane subunit of RND pump
AzCIB_1625	*czcB3-1*	1835692 - 1834538	CzcB fusion protein of RND pump
AzCIB_1626	*czcB3-2*	1836278 - 1835715	CzcB fusion protein of RND pump
AzCIB_1627	*czcC3*	1837480 - 1836275	CzcC OM export protein of RND pump
AzCIB_1685	*czcS*	1883811 - 1882480	Sensor histidine kinase
AzCIB_1686	*czcR*	1884500 - 1883814	Response regulator
AzCIB_1689	*czcC2*	1885617 - 1886879	CzcC OM export protein of RND pump
AzCIB_1690	*czcB2-2*	1886897 - 1887460	CzcB M fusion protein of RND pump
AzCIB_1691	*czcB2-1*	1887457 - 1888569	CzcB M fusion protein of RND pump
AzCIB_1692	*czcA2*	1888585 - 1891704	CzcA OM export protein of RND pump
AzCIB_1693		1891710 - 1892132	Protein conserved in *czc* clusters
AzCIB_1719		1911248 - 1910868	Protein conserved in *czc* clusters
AzCIB_1720	*czcA1*	1914416 - 1911297	CzcA OM subunit of RND pump
AzCIB_1721	*czcB1-1*	1915511 - 1914426	CzcB M fusion protein of RND pump
AzCIB_1722	*czcB1-2*	1916041 - 1915604	CzcB M fusion protein of RND pump
AzCIB_1723	*czcC1*	1917396 - 1916176	CzcC OM export protein of RND pump
AzCIB_3332		3734564 - 3735661	Cobalt transporter
AzCIB_3474	*cobS*	3887324 - 3888319	Cobalt chelation
AzCIB_3475	*cobT*	3888316 - 3890175	Cobalt chelation
AzCIB_3917	*znuC*	4371937 - 4372695	Zn transport system ATP-binding protein
AzCIB_3918	*znuB*	4372692 - 4373561	Zn transport system permease
AzCIB_3919	*znuA*	4373578 - 4374525	Zn periplasmic binding protein
**Copper (and/or silver)**			
AzCIB_0256	*copA*	0315895 - 0317316	Oxidation of Cu(I)
AzCIB_1154	*copA1*	1307039 - 1304742	Cu exporting P-type ATPase
AzCIB_1158	*copK*	1308852 - 1309247	Cu resistance protein K
AzCIB_1664		1867643 - 1867173	MerR-like transcriptional regulator
AzCIB_1665	*copA5*	1867912 - 1870152	Exporting P-type ATPase
AzCIB_1675	*cusF3*	1873739 - 1873383	Cu periplasmic binding protein
AzCIB_1676	*cusA3*	1876985 - 1873830	OM CusA protein of RND pump
AzCIB_1677	*cusB3*	1878541 - 1876982	M protein fusion CusB of RND pump

AzCIB_1678		1879862 - 1878567	OM export protein of RND pump
AzCIB_1679	*cusD3*	1880480 - 1880019	Copper, silver resistance protein
AzCIB_1702	*copK*	1896154 - 1895873	Cu resistance protein K
AzCIB_1703	*copK*	1896596 - 1896390	Cu resistance protein K
AzCIB_1705	*copK*	1897706 - 1897425	Cu resistance protein K
AzCIB_1712	*copA2*	1905239 - 1902972	Cu exporting P-type ATPase
AzCIB_1724	*cusA1*	1920689 - 1917516	CusA OM protein of RND pump
AzCIB_1725	*cusB1*	1922289 - 1920691	CusB Membrane protein of RND pump
AzCIB_1726		1923521 - 1922286	OM efflux protein of RND pump
AzCIB_1727	*cusF1*	1924019 - 1923666	Cu periplasmic binding protein of RND pump
AzCIB_1728	*cusC1*	1924434 - 1924126	Cu tolerance protein of RND pump
AzCIB_1729	*cusC1*	1924434 - 1924126	Cu tolerance protein of RND pump
AzCIB_1732	*cusR1*	1926333 - 1927013	CusR response regulator
AzCIB_1733	*cusS1*	1927010 - 1928425	CusS sensor histidine kinase
AzCIB_2191	*nosD*	2450470 - 2451768	Cu periplasmic binding protein
AzCIB_2192	*nosF*	2451740 - 2452681	ABC copper transporter
AzCIB_2193	*nosY*	2452693 - 2453508	Cu permease
AzCIB_3087	*copZ*	3462209 - 3462000	Heavy metal transport protein
AzCIB_3088	*copA3*	3464645 - 3462234	Cu exporting P-type ATPase
AzCIB_3089	*copY*	3464747 - 3465202	MerR-like transcriptional regulator
AzCIB_3108	*copA4*	3483893 - 3481365	Cu exporting P-type ATPase
AzCIB_3961	*copS2*	4418625 - 4417144	CusS sensor histidine kinase
AzCIB_3962	*copR2*	4419296 - 4418625	CusR response regulator
AzCIB_3963	*copF2*	4419452 - 4419811	Cu periplasmic binding protein
AzCIB_3964		4420037 - 4421392	OM efflux protein of RND pump
AzCIB_3965	*copB2*	4421389 - 4422927	CusB membrane protein of RND pump
AzCIB_3966	*copA2*	4423122 - 4426292	CusA OM protein of RND pump
**Chromate**			
AzCIB_2066	*chrB*	2305649 - 2306590	Chromate resistance regulatory protein
AzCIB_2067	*chrA*	2306587 - 2307951	Chromate transporter
AzCIB_3382	*chrB*	3794929 - 3795516	Chromate resistance regulatory protein
AzCIB_3383	*chrA*	3795513 - 3796064	Chromate transporter
**Mercury**			
AzCIB_1669	*merR1*	1872107 - 1871694	MerR-like transcriptional regulator
AzCIB_1670	*merT1*	1872107 - 1871694	Mercury transport protein
AzCIB_1680	*merR*	1880977 - 1880483	MerR-like transcriptional regulator
AzCIB_1681	*merR2*	1881547 - 1881134	MerR-like transcriptional regulator
AzCIB_1682	*merT2*	1881587 - 1881961	Mercury transport protein
AzCIB_1683	*merP*	1881976 - 1882263	Mercury periplasmic binding protein
**Molybdenum**			
AzCIB_4586	*modC*	5081891 - 5080785	ATPase molybdate transport system
AzCIB_4587	*modB*	5082559 - 5081888	Permease molybdate transport system
AzCIB_4588	*modA*	5083328 - 5082573	Permease molybdate transport system
**Nickel**			
AzCIB_1828	*nikC*	2043694 - 2042279	Permease transport nickel
AzCIB_1829	*nikB*	2044675 - 2043698	Permease transport nickel
AzCIB_2159	*nikE*	2405821 - 2403722	ATPase component nickel ABC transporter
**Selenium and/or tellurium**			
AzCIB_0092	*ubiE*	0111198 - 0110461	Selenite methylation
AzCIB_0135	*tpmt*	0164068 - 0163430	Tellurite methylation
AzCIB_0233	*napA*	0293956 - 0296508	Unspecific selenite reduction
AzCIB_0744	*napA2*	0834441 - 0837215	Unspecific selenite reduction
AzCIB_2072	*cysA*	2312680 - 2311613	Thiosulfate/selenite transport

AzCIB_2074	*cysT*	2314433 - 2313570	Thiosulfate/selenite permease
AzCIB_2181	*narG*	2435443 - 2439198	Unspecific selenite reduction
AzCIB_2182	*narH*	2439371 - 2440921	Unspecific selenite reduction
AzCIB_2771	*iscC*	3111558 - 3110326	Sulfur/selenium relay system
AzCIB_2771	*iscC*	3112706 - 3111555	Sulfur/selenium relay system
AzCIB_3149	*cysT*	3521058 - 3521894	Sulfate ABC transporter and Se resistance
AzCIB_3150	*cysW*	3521894 - 3522814	Sulfate ABC transporter and Se resistance
AzCIB_3151	*cysT2*	3522811 - 3523899	Sulfate transporter and Se resistance
AzCIB_3152	*cysB*	3523896 - 3524840	LysR-type sulfate regulator
AzCIB_3153	*cysK*	3524901 - 3525878	Sulfur relay protein and Se/Te resistance

TCS: two-component system
OM: outer membrane
RND: Resistance-Nodulation-Division superfamily

Adapted of Table S4 from Martín-Moldes *et al.*, 2015

TABLE S2. Oligonucleotides used in this study

Primers	Sequence (5´to 3´)	Use
5’arsC	CGGGATCCGTTCCTGTGTACCGGCAAC (BamHI)	468-bp *arsC* internal fragment cloned into double-digested pK18*mob* to generate pK18*mobarsC* used to construct CIB*darsC* mutants
3’arsC	CCCAAGCTTCAGGCCGACAGCTTGCCGA (HindIII)	468-bp *arsC* internal fragment cloned into double-digested pK18*mob* to generate pK18*mobarsC* used to construct CIB*darsC* mutants
5’ext-arsC	GGCTCTTCCAACCCTGCGC	Confirmation of the *Azoarcus* sp. d*arsC* mutants
3’ext-arsC	CATCGACACCGCACACGCC	Confirmation of the *Azoarcus* sp. d*arsC* mutants
5’arsB	CGGGATCCTGGTGAACTGGCTGGTGAAG (BamHI)	557-bp *arsB* internal fragment cloned into double-digested pK18*mob* to generate pK18*mobarsB* used to construct CIB*darsB* mutants
3’arsB	CCCAAGCTTGTAGGCGAGCGAGGAATTGA (HindIII)	557-bp *arsB* internal fragment cloned into double-digested pK18*mob* to generate pK18*mobarsB* used to construct CIB*darsB* mutants
5’ext-arsB	GTATCACAAGGAGATCGCGC	Confirmation of the *Azoarcus* sp. d*arsB* mutants
3’ext-arsB	GTCACGGTTCGTCGTCTCATTGG	Confirmation of the *Azoarcus* sp. d*arsB* mutants
5’arsC2	CGGGATCCCGATCTACCACAACCCCAAATG (BamHI)	551-bp *arsC2* internal fragment cloned into double-digested pK18*mob* to generate pK18*mobarsC2* used to construct CIB*darsC2* mutants
3’arsC2	CCCAAGCTTGTCGGCGCGGATGTTATGC (HindIII)	551-bp *arsC2* internal fragment cloned into double-digested pK18*mob* to generate pK18*mobarsC2* used to construct CIB*darsC2* mutants
5’ext-arsC2	GACACCTGCGACGACATTC	Confirmation of the *Azoarcus* sp. d*arsC2* mutants
3’ext-arsC2	AAGCGTTCAACAAGACCTG	Confirmation of the *Azoarcus* sp. d*arsC2* mutants
5’arxA	CGGGATCCGCTCACCCAACATCGGGTATCGG (BamHI)	493-bp *arxA* internal fragment cloned into double-digested pK18mob to generate pK18mobarxA used to construct CIBd*arxA* mutants
3’arxA	CCCAAGCTTCATTTCGGCGTGCGGTCTTTC (HindIII)	493-bp *arxA* internal fragment cloned into double-digested pK18mob to generate pK18mobarxA used to construct CIBd*arxA* mutants
5’ext-arxA	GACGGTTCCTCGCAGTGCATG	Confirmation of the *Azoarcus* sp. d*arxA* mutants

3’ext-arxA	CACGACGACGAAGCGCTGTG	Confirmation of the *Azoarcus* sp. d*arxA* mutants
F24	CGCCAGGGTTTTCCCAGTCACGAC	Check insertion mutant constructions in *Azoarcus* sp. CIB
R24	AGCGGATAACAATTTCACACAGGA	Check insertion mutant constructions in *Azoarcus* sp. CIB
5’RTarsC	GCTGTGCAGCAAGTCGTG	94-bp *arsC* fragment amplified in qRT-PCR
3’RTarsC	CACCACCGGACAGGTCTC	94-bp *arsC* fragment amplified in qRT-PCR
5’RTarsB	CAGTGTGAAGTCAGGGAAA	90-bp *arsB* fragment amplified in qRT-PCR
3’RTarsB	GACGATGCACAGGAACACC	90-bp *arsB* fragment amplified in qRT-PCR
5’RTarsC2	TCTACCACAACCCCAAATGC	97-bp *arsC2* fragment amplified in qRT-PCR
3’RTarsC2	GGGGTCTTCAGGTATTCGATG	97-bp *arsC2* fragment amplified in qRT-PCR
5’RTarxA	ACGAGGACGGCTACATGTTC	87-bp *arxA* fragment amplified in qRT-PCR
3’RTarxA	GTCCATCTTGTACGGCTTGC	87-bp *arxA* fragment amplified in qRT-PCR
5’POLIIIHK	CGAAACGTCGGATGCACGC	166-bp *dnaE* fragment amplified in qRT-PCR
3’POLIIIKH	GCGCAGGCCTAGGAAGTCGAAC	166-bp *dnaE* fragment amplified in qRT-PCR

TABLE S3. Operation conditions for HPLC and ICP-MS

**ICP-MS parameters for As determination**	
RF power	1550
Plasma gas flow rate (l min^{-1})	15.0
Ar Auxiliary flow rate (l min^{-1})	0.30
Carrier gas flow rate (l min^{-1})	0.75
Isotopes monitored	^{75}As
**Chromatographic parameters column**	
Stationary phase	Hamilton PRP-X100 (250mmx4.6mm,10µm)
Mobile Phase	$HPO_4^{-2}/H_2PO_4^{-1}$ (10 mM) + 2% MeOH
Mode	Isocratic
Flow rate (l min^{-1})	1.5
Injection volume (µl)	100

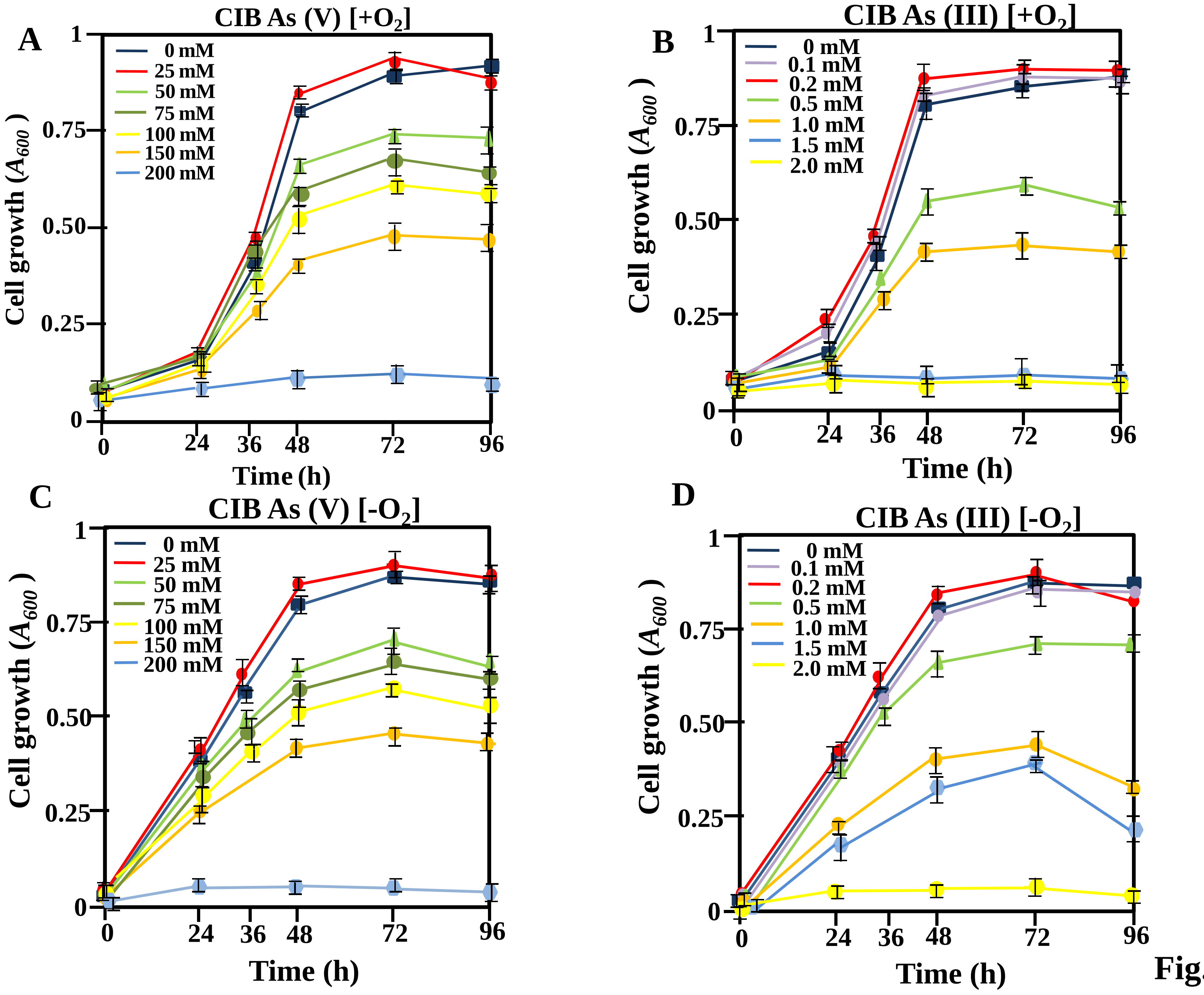
A
CIB As (V) [+O2]
0 mM
25 mM
50 mM
75 mM
100 mM
150 mM
200 mM
Cell growth (A600)
Time (h)
B
CIB As (III) [+O2]
0 mM
0.1 mM
0.2 mM
0.5 mM
1.0 mM
1.5 mM
2.0 mM
Cell growth (A600)
Time (h)
C
CIB As (V) [-O2]
0 mM
25 mM
50 mM
75 mM
100 mM
150 mM
200 mM
Cell growth (A600)
Time (h)
D
CIB As (III) [-O2]
0 mM
0.1 mM
0.2 mM
0.5 mM
1.0 mM
1.5 mM
2.0 mM
Cell growth (A600)
Time (h)

Fig. S1

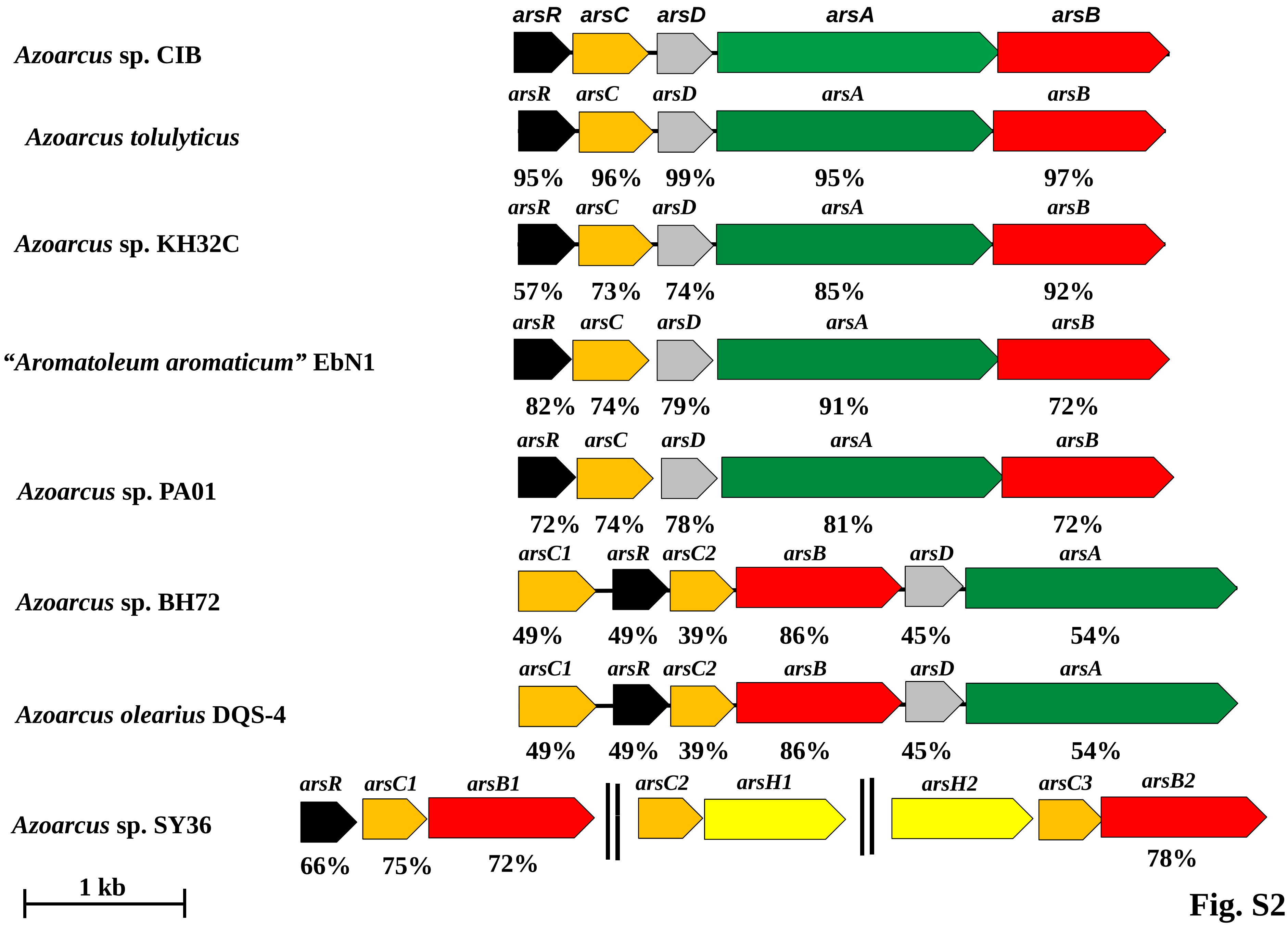

Azoarcus sp. CIB
arsR arsC arsD arsA arsB
Azoarcus tolulyticus
arsR arsC arsD arsA arsB
95% 96% 99% 95% 97%
Azoarcus sp. KH32C
arsR arsC arsD arsA arsB
57% 73% 74% 85% 92%
“Aromatoleum aromaticum” EbN1
arsR arsC arsD arsA arsB
82% 74% 79% 91% 72%
Azoarcus sp. PA01
arsR arsC arsD arsA arsB
72% 74% 78% 81% 72%
Azoarcus sp. BH72
arsC1 arsR arsC2 arsB arsD arsA
49% 49% 39% 86% 45% 54%
Azoarcus olearius DQS-4
arsC1 arsR arsC2 arsB arsD arsA
49% 49% 39% 86% 45% 54%
Azoarcus sp. SY36
arsR arsC1 arsB1 arsC2 arsH1 arsH2 arsC3 arsB2
66% 75% 72% 78%
1 kb

Fig. S2

A

```
Azoarcus sp. CIB    -------------------------MDEKAAVAVFECLSSGVRLGVFRLLVKAEPAGIVAGDIATALDIAPSSLSFHLRTMTQAGLLRVEQEGRFLRYRANLALMTEVVGFLTENCCSGV
L.ferriphilum       -------------------------MEKQAATSIFESLSSGLRLDVFRLLVKKGPGGMVAGEIANALDIPPANLSFHLKALSQAHLVTVEQEGRFQRYRADIPLMLDLIAYLTEECCSGS
A.ferridurans       ---------------------MEPLQDPAQIVARLEALASPVRLEIFRLLVEQEPTGLVSGDIAEHLGQPHNGISFHLKNLQHAGLVTVQREGRYQRYRAAMPVVRALVAYLTENCCHGT
C.testosteroni      -------------------------MKEANVIRSLSALAHEARLRVFRALVVAGPEGLTPSAIAEQLGIAPNALSFHLKELFHADLVSQERQGRNVLYRAAFPVMNELLAYLTENCCQGV
E.coli              -------------------------MPEIASLQLFKILSDETRLGIVLLLREMGE--LCVCDLCTALEQSQPKTSRHLAMLRESGLLLDRKQGKWVHYRLSPHIPSWAALVIEQAWLSQQ
P.putida            --------------MAVRAFPGGHMREILTPPIVFKCLADDTRARMTLLIAREGE--LCVCELTHALELSQPKISRHLAQLREAGILMDRRKGQWVYYRLHPEVPQWVDAMLKGVVDANQ
C.glutamicum        MTTLHTIQLANPTECCTLATGPLSSDESEHYADLFKVLGDPVRLRILSQLAAGGCGPVSVNELTDLMGLSQPTISHHLKKMTEAGFLDRVPEGRVVLHRVRPELFAELRTVLQIGSMEL-

Azoarcus sp. CIB    PGGCRDGDILSGGIPGCGSSNPARESKA--
L.ferriphilum       PDQCLELRTASKCSEEFLPLLSPTPTKATT
A.ferridurans       RDCALSGETRSPSVQEGNQ-----------
C.testosteroni      VCTSDTATSCHC------------------
E.coli              DDVQAIARKLASANCSGSGKAVCI------
P.putida            EWLSPDALRLAEMGERPQSPVACA------
C.glutamicum        ------------------------------
```

B

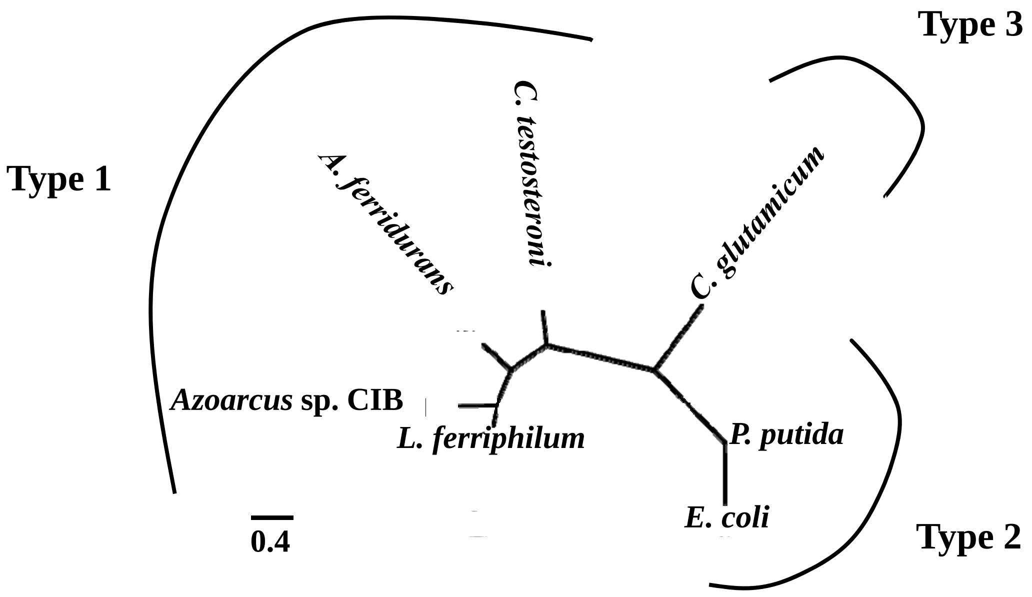

Fig. S3

A

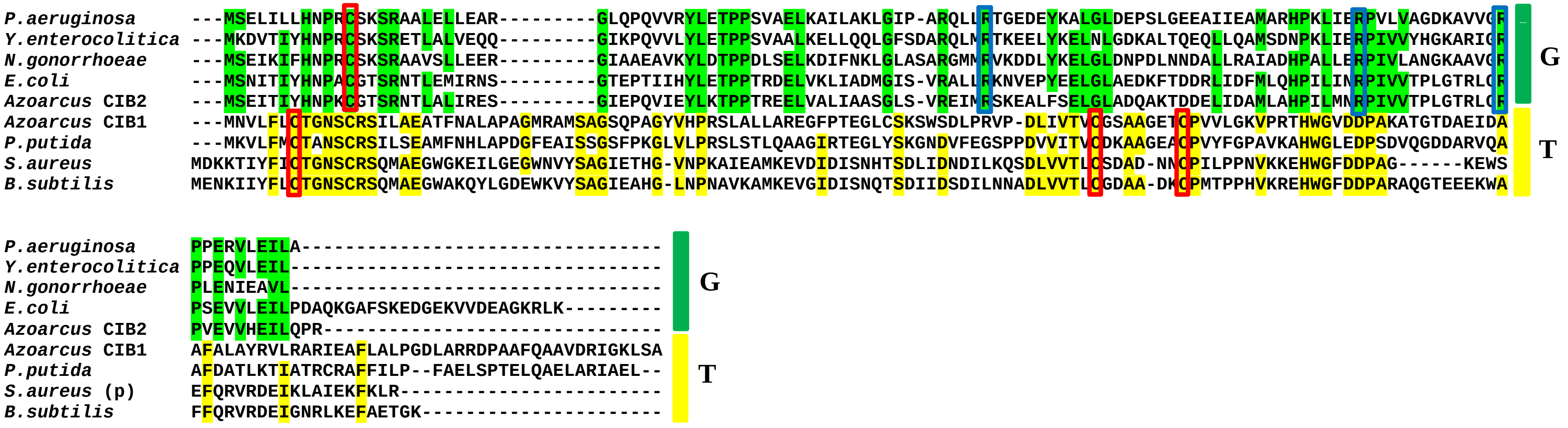
P.aeruginosa
Y.enterocolitica
N.gonorrhoeae
E.coli
Azoarcus CIB2
Azoarcus CIB1
P.putida
S.aureus
B.subtilis
G
T
P.aeruginosa
Y.enterocolitica
N.gonorrhoeae
E.coli
Azoarcus CIB2
Azoarcus CIB1
P.putida
S.aureus (p)
B.subtilis
G
T

B

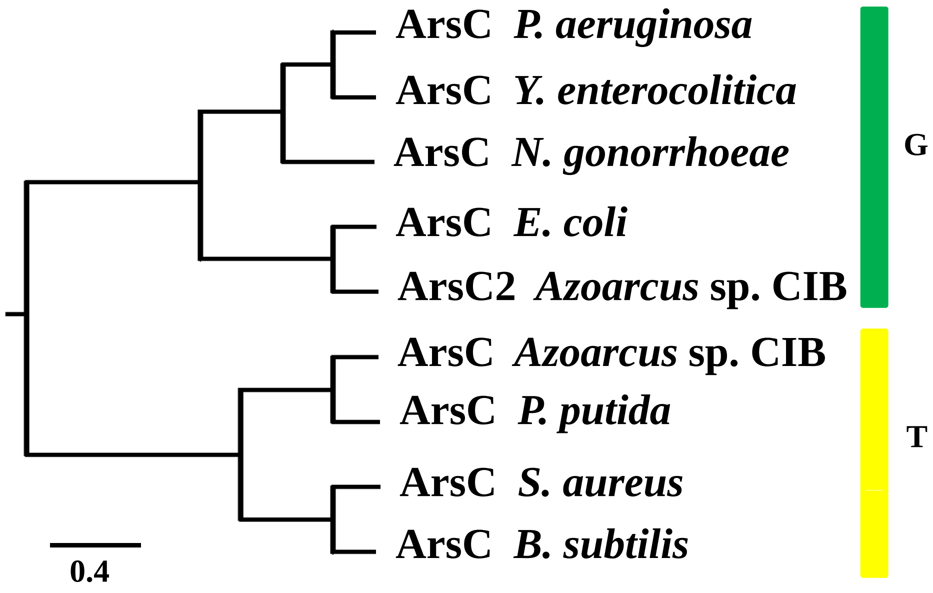
ArsC P. aeruginosa
ArsC Y. enterocolitica
ArsC N. gonorrhoeae
ArsC E. coli
ArsC2 Azoarcus sp. CIB
ArsC Azoarcus sp. CIB
ArsC P. putida
ArsC S. aureus
ArsC B. subtilis
G
T
0.4

Fig. S4

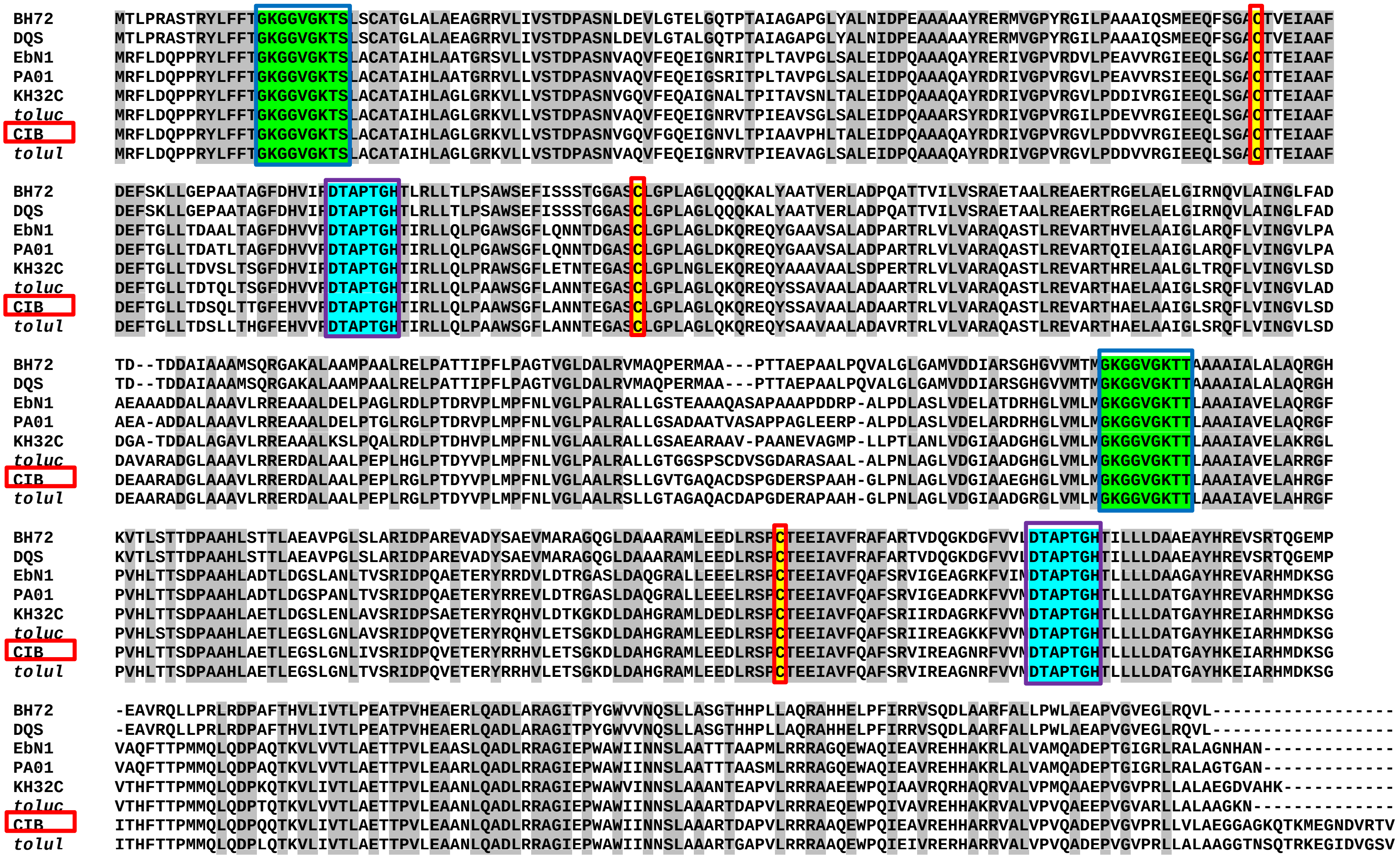
BH72 MTLPRASTRYLFFTGKGGVGKTSLSCATGLALAEAGRRVLIVSTDPASNLDEVLGTELGQTPTAIAGAPGLYALNIDPEAAAAAYRERMVGPYRGILPAAAIQSMEEQFSGACTVEIAAF
DQS MTLPRASTRYLFFTGKGGVGKTSLSCATGLALAEAGRRVLIVSTDPASNLDEVLGTALGQTPTAIAGAPGLYALNIDPEAAAAAYRERMVGPYRGILPAAAIQSMEEQFSGACTVEIAAF
EbN1 MRFLDQPPRYLFFTGKGGVGKTSLACATAIHLAATGRSVLLVSTDPASNVAQVFEQEIGNRITPLTAVPGLSALEIDPQAAAQAYRERIVGPVRDVLPEAVVRGIEEQLSGACTTEIAAF
PA01 MRFLDQPPRYLFFTGKGGVGKTSLACATAIHLAATGRRVLLVSTDPASNVAQVFEQEIGSRITPLTAVPGLSALEIDPQAAAQAYRDRIVGPVRGVLPEAVVRSIEEQLSGACTTEIAAF
KH32C MRFLDQPPRYLFFTGKGGVGKTSLACATAIHLAGLGRKVLLVSTDPASNVGQVFEQAIGNALTPITAVSNLTALEIDPQAAAQAYRDRIVGPVRGVLPDDIVRGIEEQLSGACTTEIAAF
toluc MRFLDQPPRYLFFTGKGGVGKTSLACATAIHLAGLGRKVLLVSTDPASNVAQVFEQEIGNRVTPIEAVSGLSALEIDPQAAARSYRDRIVGPVRGILPDEVVRGIEEQLSGACTTEIAAF
CIB MRFLDQPPRYLFFTGKGGVGKTSLACATAIHLAGLGRKVLLVSTDPASNVGQVFGQEIGNVLTPIAAVPHLTALEIDPQAAAQAYRDRIVGPVRGVLPDDVVRGIEEQLSGACTTEIAAF
tolul MRFLDQPPRYLFFTGKGGVGKTSLACATAIHLAGLGRKVLLVSTDPASNVAQVFEQEIGNRVTPIEAVAGLSALEIDPQAAAQAYRDRIVGPVRGVLPDDVVRGIEEQLSGACTTEIAAF
BH72 DEFSKLLGEPAATAGFDHVIIDTAPTGHTLRLLTLPSAWSEFISSSTGGASCLGPLAGLQQQKALYAATVERLADPQATTVILVSRAETAALREAERTRGELAELGIRNQVLAINGLFAD
DQS DEFSKLLGEPAATAGFDHVIIDTAPTGHTLRLLTLPSAWSEFISSSTGGASCLGPLAGLQQQKALYAATVERLADPQATTVILVSRAETAALREAERTRGELAELGIRNQVLAINGLFAD
EbN1 DEFTGLLTDAALTAGFDHVVFDTAPTGHTIRLLQLPGAWSGFLQNNTDGASCLGPLAGLDKQREQYGAAVSALADPARTRLVLVARAQASTLREVARTHVELAAIGLARQFLVINGVLPA
PA01 DEFTGLLTDATLTAGFDHVVFDTAPTGHTIRLLQLPGAWSGFLQNNTDGASCLGPLAGLDKQREQYGAAVSALADPARTRLVLVARAQASTLREVARTQIELAAIGLARQFLVINGVLPA
KH32C DEFTGLLTDVSLTSGFDHVIIDTAPTGHTIRLLQLPRAWSGFLETNTEGASCLGPLNGLEKQREQYAAAVAALSDPERTRLVLVARAQASTLREVARTHRELAALGLTRQFLVINGVLSD
toluc DEFTGLLTDTQLTSGFDHVVFDTAPTGHTIRLLQLPAAWSGFLANNTEGASCLGPLAGLQKQREQYSSAVAALADAARTRLVLVARAQASTLREVARTHAELAAIGLSRQFLVINGVLAD
CIB DEFTGLLTDSQLTTGFEHVVFDTAPTGHTIRLLQLPAAWSGFLANNTEGASCLGPLAGLQKQREQYSSAVAALADAARTRLVLVARAQASTLREVARTHAELAAIGLSRQFLVINGVLSD
tolul DEFTGLLTDSLLTHGFEHVVFDTAPTGHTIRLLQLPAAWSGFLANNTEGASCLGPLAGLQKQREQYSAAVAALADAVRTRLVLVARAQASTLREVARTHAELAAIGLSRQFLVINGVLSD
BH72 TD--TDDAIAAAMSQRGAKALAAMPAALRELPATTIPFLPAGTVGLDALRVMAQPERMAA---PTTAEPAALPQVALGLGAMVDDIARSGHGVVMTMGKGGVGKTTAAAAIALALAQRGH
DQS TD--TDDAIAAAMSQRGAKALAAMPAALRELPATTIPFLPAGTVGLDALRVMAQPERMAA---PTTAEPAALPQVALGLGAMVDDIARSGHGVVMTMGKGGVGKTTAAAAIALALAQRGH
EbN1 AEAAADDALAAAVLRREAAALDELPAGLRDLPTDRVPLMPFNLVGLPALRALLGSTEAAAQASAPAAAPDDRP-ALPDLASLVDELATDRHGLVMLMGKGGVGKTTLAAAIAVELAQRGF
PA01 AEA-ADDALAAAVLRREAAALDELPTGLRGLPTDRVPLMPFNLVGLPALRALLGSADAATVASAPPAGLEERP-ALPDLASLVDELARDRHGLVMLMGKGGVGKTTLAAAIAVELAQRGF
KH32C DGA-TDDALAGAVLRREAAALKSLPQALRDLPTDHVPLMPFNLVGLAALRALLGSAEARAAV-PAANEVAGMP-LLPTLANLVDGIAADGHGLVMLMGKGGVGKTTLAAAIAVELAKRGL
toluc DAVARADGLAAAVLRRERDALAALPEPLHGLPTDYVPLMPFNLVGLPALRALLGTGGSPSCDVSGDARASAAL-ALPNLAGLVDGIAADGHGLVMLMGKGGVGKTTLAAAIAVELARRGF
CIB DEAARADGLAAAVLRRERDALAALPEPLRGLPTDYVPLMPFNLVGLAALRSLLGVTGAQACDSPGDERSPAAH-GLPNLAGLVDGIAAEGHGLVMLMGKGGVGKTTLAAAIAVELAHRGF
tolul DEAARADGLAAAVLRRERDALAALPEPLRGLPTDYVPLMPFNLVGLAALRSLLGTAGAQACDAPGDERAPAAH-GLPNLAGLVDGIAADGRGLVMLMGKGGVGKTTLAAAIAVELAHRGF
BH72 KVTLSTTDPAAHLSTTLAEAVPGLSLARIDPAREVADYSAEVMARAGQGLDAAARAMLEEDLRSPCTEEIAVFRAFARTVDQGKDGFVVLDTAPTGHTILLLDAAEAYHREVSRTQGEMP
DQS KVTLSTTDPAAHLSTTLAEAVPGLSLARIDPAREVADYSAEVMARAGQGLDAAARAMLEEDLRSPCTEEIAVFRAFARTVDQGKDGFVVLDTAPTGHTILLLDAAEAYHREVSRTQGEMP
EbN1 PVHLTTSDPAAHLADTLDGSLANLTVSRIDPQAETERYRRDVLDTRGASLDAQGRALLEEELRSPCTEEIAVFQAFSRVIGEAGRKFVIMDTAPTGHTLLLLDAAGAYHREVARHMDKSG
PA01 PVHLTTSDPAAHLADTLDGSPANLTVSRIDPQAETERYRREVLDTRGASLDAQGRALLEEELRSPCTEEIAVFQAFSRVIGEADRKFVVMDTAPTGHTLLLLDATGAYHREVARHMDKSG
KH32C PVHLTTSDPAAHLAETLDGSLENLAVSRIDPSAETERYRQHVLDTKGKDLDAHGRAMLDEDLRSPCTEEIAVFQAFSRIIRDAGRKFVVMDTAPTGHTLLLLDATGAYHREIARHMDKSG
toluc PVHLSTSDPAAHLAETLEGSLGNLAVSRIDPQVETERYRQHVLETSGKDLDAHGRAMLEEDLRSPCTEEIAVFQAFSRIIREAGKKFVVMDTAPTGHTLLLLDATGAYHKEIARHMDKSG
CIB PVHLTTSDPAAHLAETLEGSLGNLIVSRIDPQVETERYRRHVLETSGKDLDAHGRAMLEEDLRSPCTEEIAVFQAFSRVIREAGNRFVVMDTAPTGHTLLLLDATGAYHKEIARHMDKSG
tolul PVHLTTSDPAAHLAETLEGSLGNLTVSRIDPQVETERYRRHVLETSGKDLDAHGRAMLEEDLRSPCTEEIAVFQAFSRVIREAGNRFVVMDTAPTGHTLLLLDATGAYHKEIARHMDKSG
BH72 -EAVRQLLPRLRDPAFTHVLIVTLPEATPVHEAERLQADLARAGITPYGWVVNQSLLASGTHHPLLAQRAHHELPFIRRVSQDLAARFALLPWLAEAPVGVEGLRQVL-----------------
DQS -EAVRQLLPRLRDPAFTHVLIVTLPEATPVHEAERLQADLARAGITPYGWVVNQSLLASGTHHPLLAQRAHHELPFIRRVSQDLAARFALLPWLAEAPVGVEGLRQVL-----------------
EbN1 VAQFTTPMMQLQDPAQTKVLVVTLAETTPVLEAASLQADLRRAGIEPWAWIINNSLAATTTAAPMLRRRAGQEWAQIEAVREHHAKRLALVAMQADEPTGIGRLRALAGNHAN-------------
PA01 VAQFTTPMMQLQDPAQTKVLVVTLAETTPVLEAARLQADLRRAGIEPWAWIINNSLAATTTAASMLRRRAGQEWAQIEAVREHHAKRLALVAMQADEPTGIGRLRALAGTGAN-------------
KH32C VTHFTTPMMQLQDPKQTKVLIVTLAETTPVLEAANLQADLRRAGIEPWAWVINNSLAAANTEAPVLRRRAAEEWPQIAAVRQRHAQRVALVPMQAAEPVGVPRLLALAEGDVAHK-----------
toluc VTHFTTPMMQLQDPTQTKVLVVTLAETTPVLEAANLQADLRRAGIEPWAWIINNSLAAARTDAPVLRRRAEQEWPQIVAVREHHAKRVALVPVQAEEPVGVARLLALAAGKN-------------
CIB ITHFTTPMMQLQDPQQTKVLIVTLAETTPVLEAANLQADLRRAGIEPWAWIINNSLAAARTDAPVLRRRAAQEWPQIEAVREHHARRVALVPVQADEPVGVPRLLVLAEGGAGKQTKMEGNDVRTV
tolul ITHFTTPMMQLQDPLQTKVLIVTLAETTPVLEAANLQADLRRAGIEPWAWIINNSLAAARTGAPVLRRRAAQEWPQIEIVRERHARRVALVPVQADEPVGVPRLLALAAGGTNSQTRKEGIDVGSV

Fig. S5

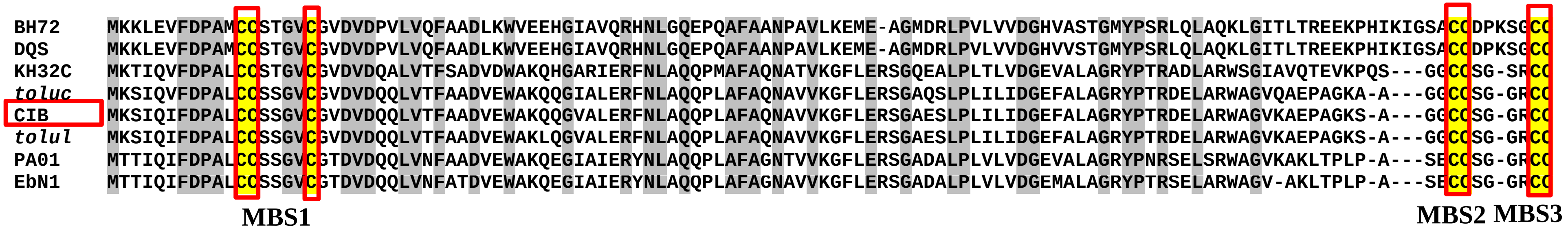
BH72 MKKLEVFDPAMCCSTGVCGVDVDPVLVQFAADLKWVEEHGIAVQRHNLGQEPQAFAANPAVLKEME-AGMDRLPVLVVDGHVASTGMYPSRLQLAQKLGITLTREEKPHIKIGSACCDPKSGCC
DQS MKKLEVFDPAMCCSTGVCGVDVDPVLVQFAADLKWVEEHGIAVQRHNLGQEPQAFAANPAVLKEME-AGMDRLPVLVVDGHVVSTGMYPSRLQLAQKLGITLTREEKPHIKIGSACCDPKSGCC
KH32C MKTIQVFDPALCCSTGVCGVDVDQALVTFSADVDWAKQHGARIERFNLAQQPMAFAQNATVKGFLERSGQEALPLTLVDGEVALAGRYPTRADLARWSGIAVQTEVKPQS---GGCCSG-SRCC
toluc MKSIQVFDPALCCSSGVCGVDVDQQLVTFAADVEWAKQQGIALERFNLAQQPLAFAQNAVVKGFLERSGAQSLPLILIDGEFALAGRYPTRDELARWAGVQAEPAGKA-A---GGCCSG-GRCC
CIB MKSIQIFDPALCCSSGVCGVDVDQQLVTFAADVEWAKQQGVALERFNLAQQPLAFAQNAVVKGFLERSGAESLPLILIDGEFALAGRYPTRDELARWAGVKAEPAGKS-A---GGCCSG-GRCC
tolul MKSIQIFDPALCCSSGVCGVDVDQQLVTFAADVEWAKLQGVALERFNLAQQPLAFAQNAVVKGFLERSGAESLPLILIDGEFALAGRYPTRDELARWAGVKAEPAGKS-A---GGCCSG-GRCC
PA01 MTTIQIFDPALCCSSGVCGTDVDQQLVNFAADVEWAKQEGIAIERYNLAQQPLAFAGNTVVKGFLERSGADALPLVLVDGEVALAGRYPNRSELSRWAGVKAKLTPLP-A---SECCSG-GRCC
EbN1 MTTIQIFDPALCCSSGVCGTDVDQQLVNFATDVEWAKQEGIAIERYNLAQQPLAFAGNAVVKGFLERSGADALPLVLVDGEMALAGRYPTRSELARWAGV-AKLTPLP-A---SECCSG-GRCC
MBS1
MBS2
MBS3

Fig. S6

```
Azoarcus BH72(1)      -MTPLKTVLVLCTGNSCRSQMAEAILNHDLAGRVHALSAGTVPQPKVADGAIEALKLAGLPTAGLFPKDVDAVLN---QPIDLVVTVCDNA-KESCPVFPRPVPRIHQPFHDPHGEPLES
Azoarcus BH72(2)      MSERIYHVLFLCTGNSARSILAESILNQLGHGRFVAHSAGSHPKGEVHPLALELLERNHLPTAGLRSKSWDEFAAPGAPALDFVFTVCDNAAGEVCPVWPGQPMTAHWGLEDPAEAEGDE
Azoarcus KH32C        -----MNVLFLCTGNSCRSILGEATFNHLAPAGWKAMSAGSKPAGYVHPRSIALLAREGLPTEGYYSKSWDKLPA----MPDIVITVCGSAAGETCPAYLGPVLRTHWGVEDPAHATGTD
A.aromaticum EbN1     -----MKVLFLCTGNSCRSILAEATFNALAPHGLEAISAGSRPAGYVHPRALALLAREGISTEGLSSKSWDGLPV----TPDIVITVCGSAASETCPAYLGPVVRAHWGVEDPAQASGSD
Azoarcus PA01         -----MKVLFLCTGNSCRSILAEATFNALAPHRLKALSAGSRPAGYVHPRALALLAREGISTEGLSSKAWDGLPG----TPDVVITVCGSAASETCPAYLGPAVRAHWGVEDPAGASGSD
Azoarcus CIB         -----MNVLFLCTGNSCRSILAEATFNALAPAGMRAMSAGSQPAGYVHPRSLALLAREGFPTEGLCSKSWSDLPR----VPDLIVTVCGSAAGETCPVVLGKVPRTHWGVDDPAKATGTD
A.toluclasticus       -----MNVLFLCTGNSCRSILAEATFNALAPAGMRAMSAGSQPAGYVHPRSLALLAREGIATEGVHSKSWNGLPH----TPDLVVTVCASAAGETCPVYLGKVSRTHWGVADPARATGSD

Azoarcus BH72(1)      ------FVAVRDDIRARLVPAVRAALGL--------------------
Azoarcus BH72(2)      AA-RKAMSAALRLLSHRIGLFLSLPLAK--LDRLSLQSQLQGIGREAG
Azoarcus KH32C        EEIDAAFMDAYHILRARIEAFLALPLEELKQDRARLKVEMDRIGTLIK
A.aromaticum EbN1     ARIDAAFEHAYRTLRARIEAFFALPENLARRDPPAFRAGLERIGHIAA
Azoarcus PA01         ARIDAAFDHAYRTLRARIEAFLALPENLARRDPAAFRAALERIGHIAA
Azoarcus CIB          AEIDAAFALAYRVLRARIEAFLALPGDLARRDPAAFQAAVDRIGKLSA
A.toluclasticus       AEIDAAFEHAYRVLRARIEAFLALPGELARRDPVAFQSEVDRIGTLLP
```

Fig. S7

```
QS      ---METLNAAELLAALGHETRLSIFRLLVEAGPAGLNASAIGEHLSLAPATLSFHLAHLSRVGLIVGERESRFIHYSARFDIMDELIAFLTRNCCQGSACLPKT----------------TGCDTTAKRRAATDKDSA
BH72    ---METLNAAELLAALGHETRLSIFRLLVEAGPAGLNASAIGEHLSLAPATLSFHLAHLSRVGLIVGERESRFIHYSARFDTMDELIAFLTRNCCQGSACLPKT----------------AGCDTTAKRRAATDKDSA
PA01    ---MDQKSALAVFESLSSGVRLDVFRLLVKAEPTGIVAGEIASALDVAPSSLSFHLRTLTQAGLLTVEQEGRFLRYRANLPLMAEVIGFLTENCCAGVPGRCVAVCDVEAQL----ADCSGSCGTGTESRA-------
EbN1    ---MDQKSALAVFESLSSGVRLDVFRLLVKAEPTGVVAGEIASALDVAPSSLSFHLRTLTQAGLLTVEQEGRFLRYRANLSLMAEVIGFLTENCCAGVPERCAAVCDVEAQL----ADCSGSRGTGTEPRA-------
CIB     ---MDEKAAVAVFECLSSGVRLGVFRLLVKAEPAGIVAGDIATALDIAPSSLSFHLRTMTQAGLLRVEQEGRFLRYRANLALMTEVVGFLTENCCSGVPGGCRDGDILSGGI----PGCGSSNPA-RESKA-------
tolul   ---MDEKAAVAVFECLSSGVRLGVFRLLVKAEPVGIVAGDIATALDIAPSSLSFHLRTMTQAGLLRVEQEGRFLRYRANLGLMSEVVGFLTENCCSGVAGGCRDADILSGRI----PGCGSSNPA-RESKA-------
tolu    ---MDEKAALAVFECLSSGVRLGVFRLLVKAEPVGVVAGDIAAALDVAPSSLSFHLRTMTQAGLLRVEQEGRFLRYRANLALMTEVVGFLTANCCSGVPGGCRDADVLAGRI----SDCGASNPM-QESKA-------
SY39    ---MDKPTALQVFEALSSGVRLDVFRLLVQAGPDGRVAGELSAALDIPPTNLSFHLKGLAQAGLVTAEQEGRFQRYRANIALMVDTIGFLTENCCGEGVSCE----------------LTGIAEGVCKS---------
KH32C   MSTIQKDQVLRVFEALSSGVRLDVFRLLVQAGNGGRVAGEIGAALDIPPTNLSFHLKALVQAGLLSVEQEGRYQRYRASIPLMLDTIAYLTANCCGDHPEECAEYRDRVPALDGFLPPLSCSADGGCKK---------
```

Fig. S8

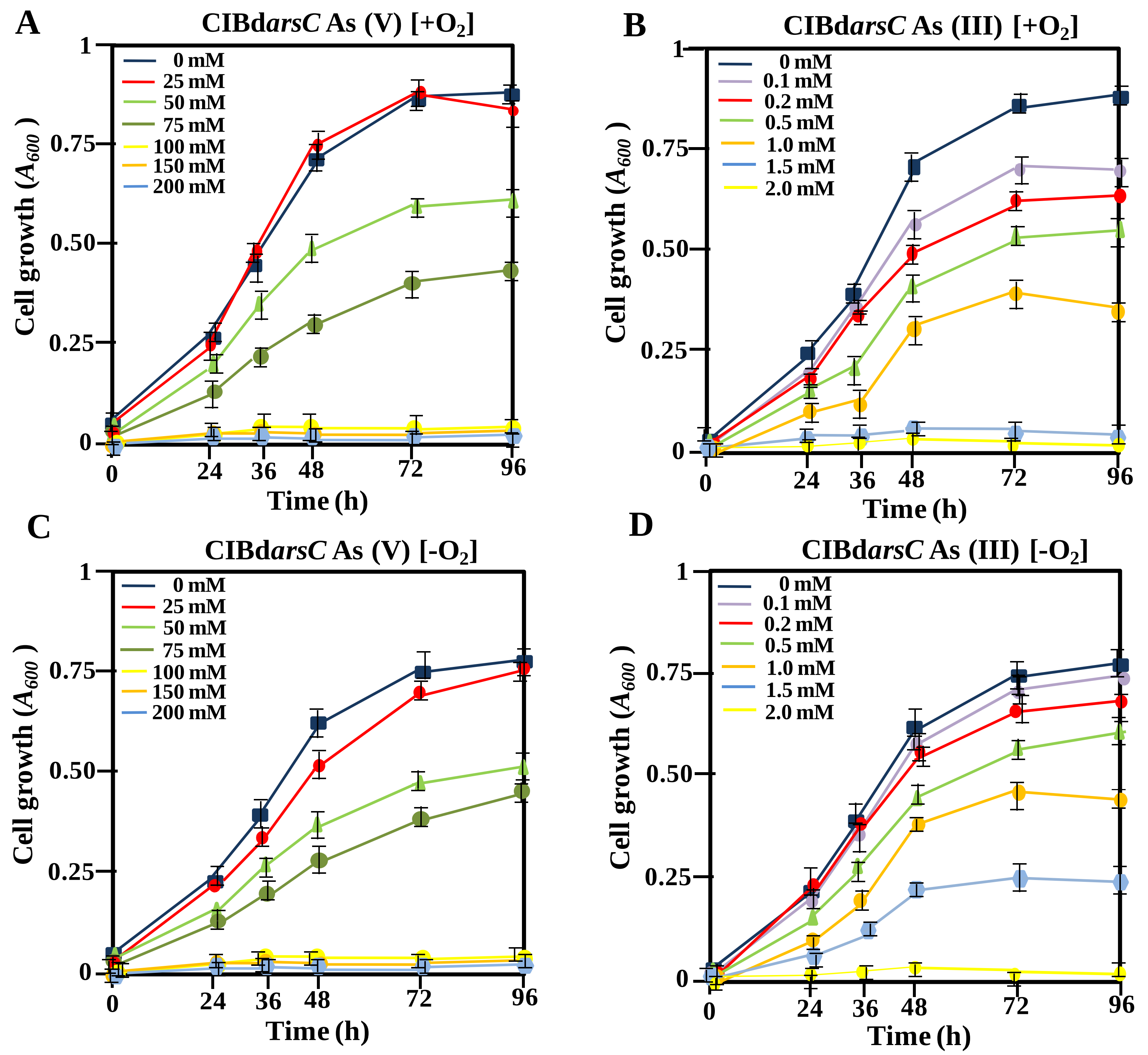
A
CIBd*arsC* As (V) [+O2]
B
CIBd*arsC* As (III) [+O2]
C
CIBd*arsC* As (V) [-O2]
D
CIBd*arsC* As (III) [-O2]
0 mM
25 mM
50 mM
75 mM
100 mM
150 mM
200 mM
0.1 mM
0.2 mM
0.5 mM
1.0 mM
1.5 mM
2.0 mM
Cell growth (A600)
Time (h)
0
24
36
48
72
96
0.25
0.50
0.75
1

Fig. S9

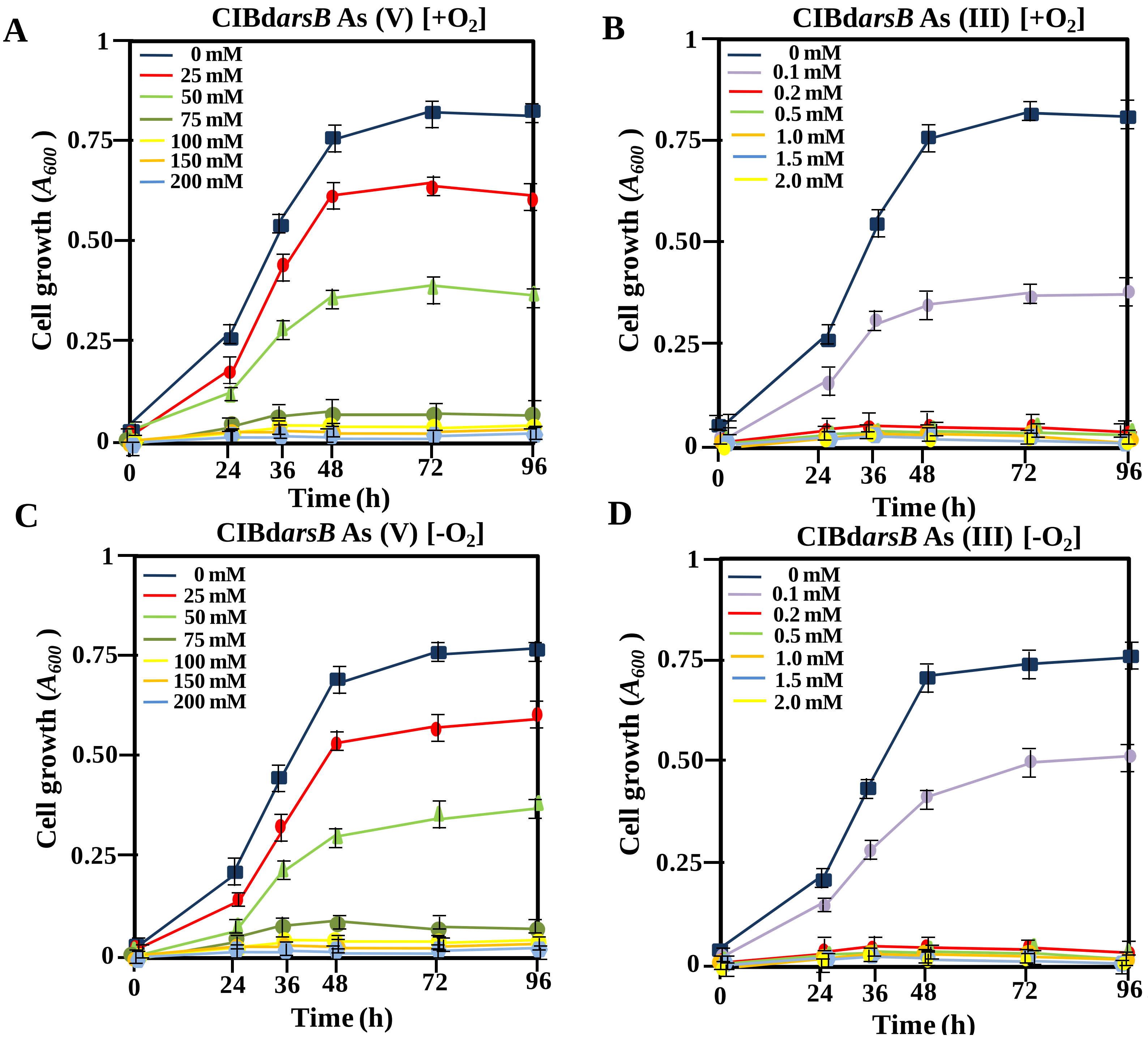

A
CIBd*arsB* As (V) [+O_2]
0 mM
25 mM
50 mM
75 mM
100 mM
150 mM
200 mM
Cell growth (A_{600})
1
0.75
0.50
0.25
0
0
24
36
48
72
96
Time (h)
B
CIBd*arsB* As (III) [+O_2]
0 mM
0.1 mM
0.2 mM
0.5 mM
1.0 mM
1.5 mM
2.0 mM
Cell growth (A_{600})
1
0.75
0.50
0.25
0
0
24
36
48
72
96
Time (h)
C
CIBd*arsB* As (V) [-O_2]
0 mM
25 mM
50 mM
75 mM
100 mM
150 mM
200 mM
Cell growth (A_{600})
1
0.75
0.50
0.25
0
0
24
36
48
72
96
Time (h)
D
CIBd*arsB* As (III) [-O_2]
0 mM
0.1 mM
0.2 mM
0.5 mM
1.0 mM
1.5 mM
2.0 mM
Cell growth (A_{600})
1
0.75
0.50
0.25
0
0
24
36
48
72
96
Time (h)

Fig. S10

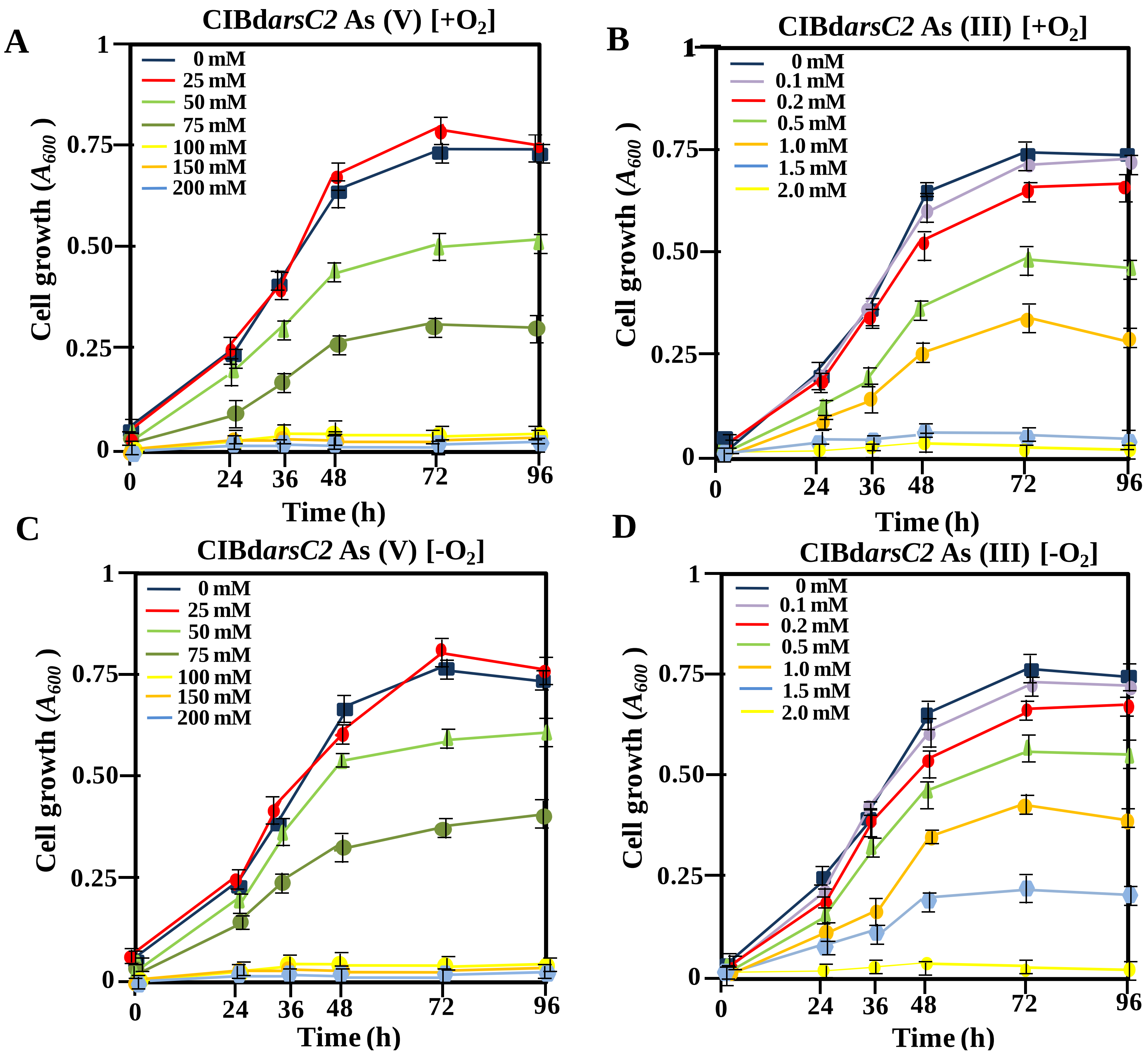
A
CIBd*arsC2* As (V) [+O_2]
0 mM
25 mM
50 mM
75 mM
100 mM
150 mM
200 mM
Cell growth (A_{600})
Time (h)
B
CIBd*arsC2* As (III) [+O_2]
0 mM
0.1 mM
0.2 mM
0.5 mM
1.0 mM
1.5 mM
2.0 mM
Cell growth (A_{600})
Time (h)
C
CIBd*arsC2* As (V) [-O_2]
0 mM
25 mM
50 mM
75 mM
100 mM
150 mM
200 mM
Cell growth (A_{600})
Time (h)
D
CIBd*arsC2* As (III) [-O_2]
0 mM
0.1 mM
0.2 mM
0.5 mM
1.0 mM
1.5 mM
2.0 mM
Cell growth (A_{600})
Time (h)

Fig. S11

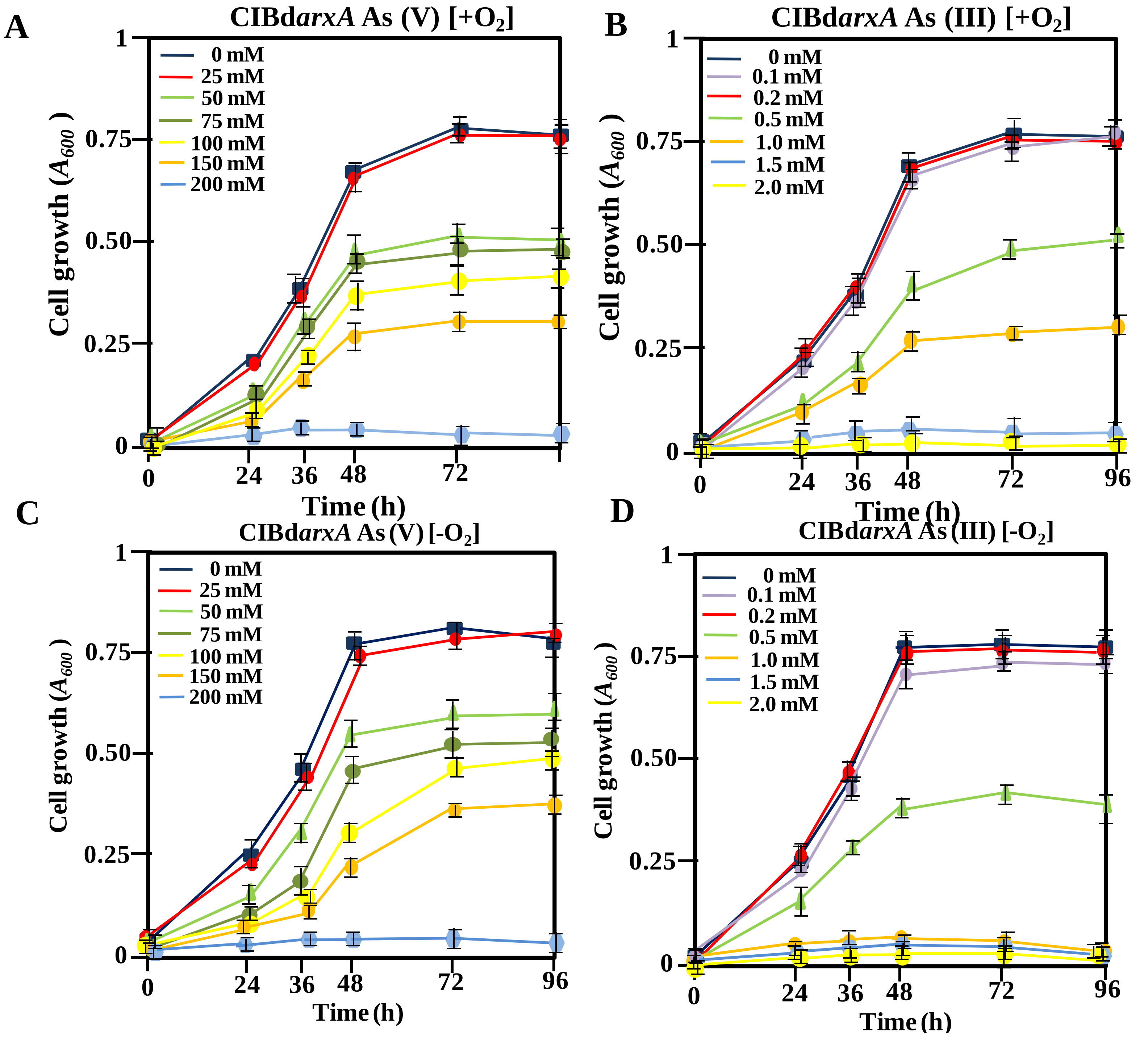
A
CIBd*arxA* As (V) [+O2]
B
CIBd*arxA* As (III) [+O2]
C
CIBd*arxA* As (V) [-O2]
D
CIBd*arxA* As (III) [-O2]
Cell growth (A600)
Time (h)
0 mM
25 mM
50 mM
75 mM
100 mM
150 mM
200 mM
0.1 mM
0.2 mM
0.5 mM
1.0 mM
1.5 mM
2.0 mM
0
0.25
0.50
0.75
1
24
36
48
72
96

Fig. S12

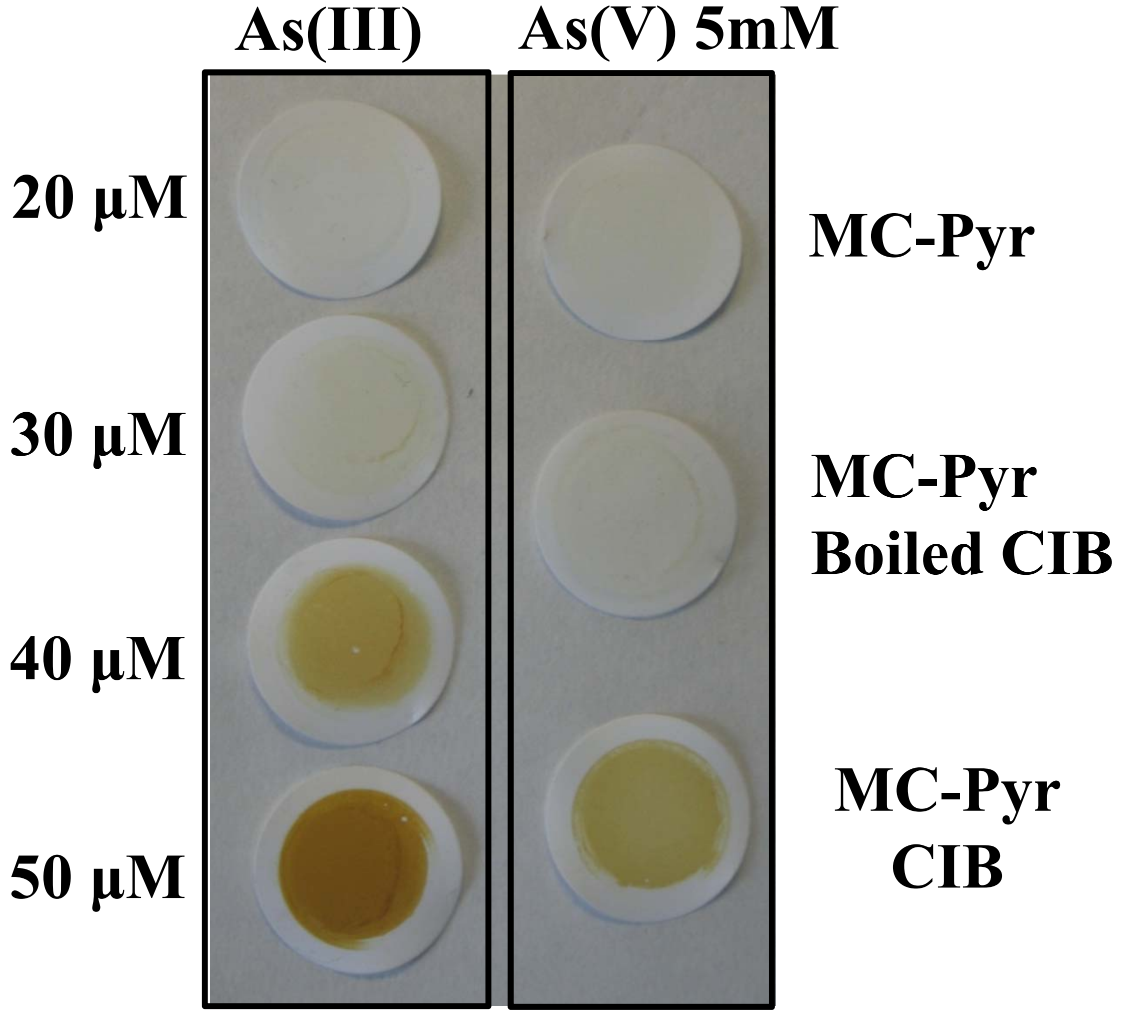

As(III)
As(V) 5mM
20 μM
30 μM
40 μM
50 μM
MC-Pyr
MC-Pyr
Boiled CIB
MC-Pyr
CIB

Fig. S13

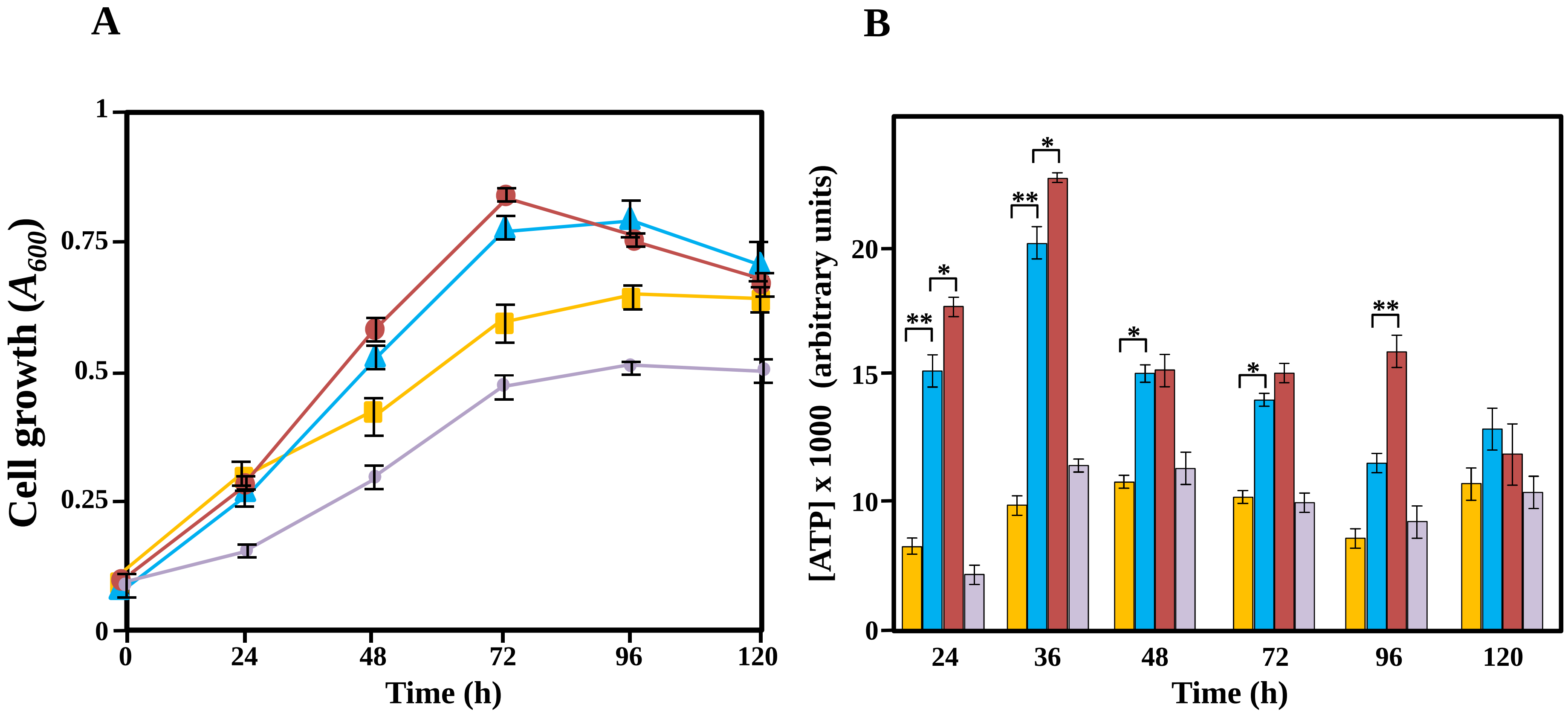

Fig. S14

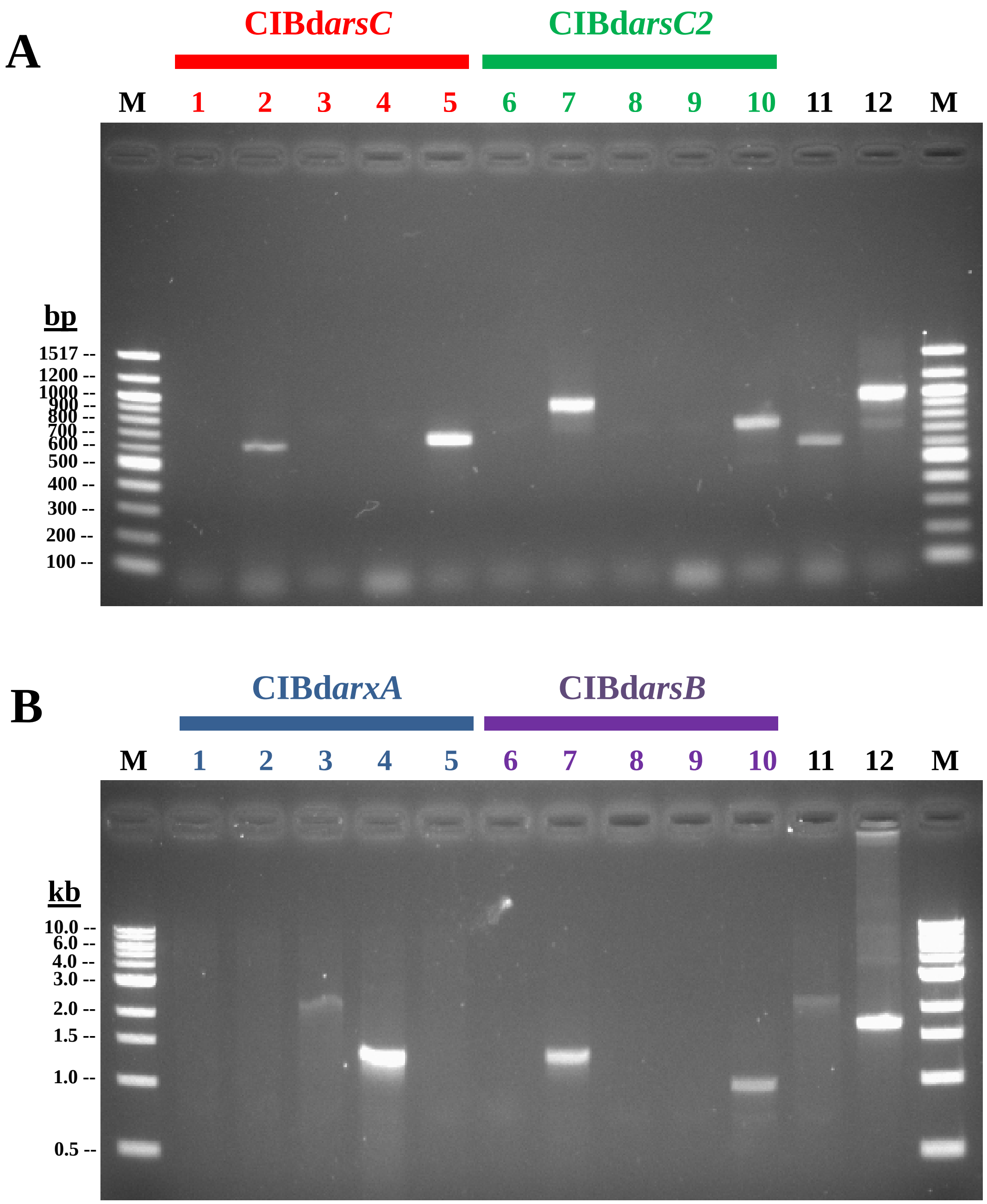
A
CIBdarsC
CIBdarsC2
M 1 2 3 4 5 6 7 8 9 10 11 12 M
bp
1517 --
1200 --
1000 --
900 --
800 --
700 --
600 --
500 --
400 --
300 --
200 --
100 --
B
CIBdarxA
CIBdarsB
M 1 2 3 4 5 6 7 8 9 10 11 12 M
kb
10.0 --
6.0 --
4.0 --
3.0 --
2.0 --
1.5 --
1.0 --
0.5 --

Fig. S15
